# Layperson-Led vs Professional-Led Behavioral Interventions for Weight Loss in Pediatric Obesity: A Systematic Review and Meta-analysis

**DOI:** 10.1001/jamanetworkopen.2020.10364

**Published:** 2020-07-13

**Authors:** Jonathan McGavock, Bhupendrasinh F. Chauhan, Rasheda Rabbani, Sofia Dias, Nika Klaprat, Sara Boissoneault, Justin Lys, Aleksandra K. Wierzbowski, Mohammad Nazmus Sakib, Ryan Zarychanski, Ahmed M. Abou-Setta

**Affiliations:** 1 Children's Hospital Research Institute of Manitoba, Winnipeg, Canada; 2 Department of Pediatrics and Child Health, University of Manitoba, Winnipeg, Canada; 3 Diabetes Research Envisioned and Accomplished in Manitoba, Children’s Hospital Research Institute of Manitoba, Winnipeg, Canada; 4 George & Fay Yee Centre for Healthcare Innovation, Winnipeg, Manitoba, Canada; 5 I. H. Asper Clinical Research Institute, St. Boniface Hospital Research Centre, Winnipeg, Manitoba, Canada; 6 Centre for Reviews and Dissemination, University of York, York, United Kingdom; 7 School of Public Health and Health Systems, University of Waterloo, Waterloo, Ontario, Canada; 8 Department of Haematology and Medical Oncology, CancerCare Manitoba, Winnipeg, Canada; 9 Department of Internal Medicine, University of Manitoba, Winnipeg, Canada

## Abstract

**Importance:**

The appropriate approach for weight loss among children and adolescents with overweight and obesity remains unclear.

**Objective:**

To evaluate the difference in the treatment outcomes associated with behavioral weight loss interventions led by laypersons and professionals in comparison with unsupervised control arms among children and adolescents with overweight and obesity.

**Data Sources:**

For this systematic review and meta-analysis, the Medical Literature Analysis and Retrieval System Online (MEDLINE), Embase, the Cochrane Library, and Cumulative Index of Nursing and Allied Health Literature (CINAHL) databases were searched from January 1, 1996, to June 1, 2019.

**Study Selection:**

Included in this study were randomized clinical trials (RCTs) of behavioral interventions lasting at least 12 weeks for children and adolescents (aged 5-18 years) with overweight and obesity. Exclusion criteria included non-RCT studies, interventions lasting less than 12 weeks, adult enrollment, participants with other medical diagnoses, pharmacological treatment use, and articles not written in English. Two of 6 reviewers independently screened all citations. Of 25 586 citations, after duplicate removal, 78 RCTs (5780 participants) met eligibility criteria.

**Data Extraction and Synthesis:**

A bayesian framework and Markov chain Monte Carlo simulation methods were used to combine direct and indirect associations. Random-effects and fixed-effect network meta-analysis models were used with the preferred model chosen by comparing the deviance information criteria. This study was registered with the International Prospective Register of Systematic Reviews (PROSPERO) and followed the Preferred Reporting Items for Systematic Reviews and Meta-analyses (PRISMA) reporting guideline.

**Main Outcomes and Measures:**

The immediate and sustained changes in weight and body mass index (BMI) standardized mean difference (SMD) were primary outcomes planned before data collection began, whereas waist circumference and percent body fat were secondary outcomes. The hypothesis being tested was formulated before the data collection.

**Results:**

Of 25 586 citations retrieved, we included 78 RCTs (5780 participants), with a follow-up of 12 to 104 weeks. Compared with the control condition, random-effects models revealed that professional-led weight loss interventions were associated with reductions in weight (mean difference [MD], −1.60 kg [95% CI, −2.30 to −0.99 kg]; 68 trials; *P* < .001) and BMI (SMD, −0.30 [95% CI, −0.39 to −0.20]; 59 trials; *P* < .001) that were not sustained long term (weight MD, −1.02 kg [95% CI, −2.20 to 0.34 kg]; 21 trials; *P* = .06; BMI SMD, −0.12 [95% CI, −0.46 to 0.21]; 20 trials; *P* < .001). There was no association between layperson-led interventions and weight loss in the short-term (MD, −1.40 kg [95% CI, −3.00 to 0.26 kg]; 5 trials; *P* = .05) or long-term (MD, −0.98 kg [95% CI, −3.60 to 1.80 kg]; 1 trial; *P* = .23) compared with standard care. No difference was found in head-to-head trials (professional vs layperson MD, −0.25 kg [95% CI −1.90 to 1.30 kg]; 5 trials; *P* = .38).

**Conclusions and Relevance:**

This systematic review and meta-analysis found that professional-led weight loss interventions were associated with short-term but not sustained weight reduction among children and adolescents with overweight or obesity, and the evidence for layperson-led approaches was insufficient to draw firm conclusions.

## Key Points

**Question:**

What are the short- and long-term associations of professional- and layperson-led behavioral interventions with weight loss for children and adolescents with overweight and obesity?

**Findings:**

In this network meta-analysis of 78 unique clinical trials including 5780 participants, professional-led, but not layperson-led, interventions were associated with short-term reductions in absolute and relative weight compared with standard care. These reductions were not sustained long term.

**Meaning:**

Professional-led behavioral weight loss interventions were associated with weight reduction among children and adolescents with overweight and obesity; there was a lack of direct evidence for the association of layperson-led approaches.

## Teaser

This systematic review and meta-analysis investigates the association of professional-led and layperson-led weight loss interventions with short- and long-term weight reduction among children and adolescents with overweight and obesity.

## Introduction

Child and adolescent obesity are a global public health concern.^1^ Intensive behavioral lifestyle therapy is considered the cornerstone for treatment of obesity in this age group.^1,2^ Systematic reviews of the literature show that intensive behavioral lifestyle interventions elicit modest short-term weight loss^3,4^ and improved cardiometabolic health^4,5^ among children and adolescents with overweight and obesity. While efficacious, these approaches are costly and often impractical in real-world settings. Less intensive interventions delivered in community settings are less costly but often yield less significant weight loss.^6,7,8^ Few studies have directly compared the association between short-term and sustained interventions on weight management in children and adolescents with overweight and obesity.^9,10,11^

Engaging laypersons or community-based health workers to deliver health interventions is an attractive public health model for disease management, as it is cost-effective and can be tailored to local needs.^12,13^ In some settings, community health workers or peer leaders yield meaningful improvements in lifestyle behaviors and health outcomes among persons living with chronic disease.^14,15^ A series of recent trials suggested that behavioral interventions led by nonprofessionals yield similar results to those led by trained professionals.^7,8^ In the context of pediatric obesity, a limited number of trials suggest that peer- or layperson-led approaches may be associated with the achievement of successful weight loss.^6,7,8^ To the best of our knowledge, this has yet to be investigated by a systematic literature review with meta-analysis.

Network meta-analysis allows for the comparison of multiple treatments in 1 statistical model.^16^ Network meta-analyses can assess treatment outcomes or safety when few direct head-to-head trials exist.^17,18^ With the abundance of therapeutic trials for weight loss among children and adolescents with overweight or obesity,^2^ a network meta-analysis is an attractive model for comparing the associations of layperson- and professional-led approaches with weight loss. Accordingly, we conducted a systematic review and network meta-analysis to assess the association of behavioral interventions led by lay individuals vs those led by professionals, compared with the standard of care, with short- and long-term weight loss among children and adolescents younger than 18 years with overweight or obesity.

## Methods

### Data Sources, Search Strategy, and Eligibility Criteria

This review was conducted according to the Methodological Expectations of Cochrane Interventional Reviews (MECIR) and reported according to the Preferred Reporting Items for Systematic Reviews and Meta-analyses (PRISMA) reporting guideline. We searched the Medical Literature Analysis and Retrieval System Online (MEDLINE), Ovid (Wolters Kluwer Health), Embase Ovid (Wolters Kluwer Health), Cumulative Index of Nursing and Allied Health Literature (CINAHL), EBSCOhost (EBSCO Information Services), and the Cochrane Library (Wiley) databases. A combination of controlled vocabulary (eg, weight, intervention) and keywords (eg, overweight, obese, child, youth, and adolescents), in addition to free-text terms, were used. A search for randomized clinical trials (RCTs) from January 1, 1996, to June 1, 2019, was conducted without any restriction on the language of publication (eTable 1 in the Supplement). The systematic review followed a priori eligibility criteria, and the protocol was registered on PROSPERO (CRD:42017052977). As determined by the Biomedical Research Ethics Board at the University of Manitoba, institutional ethics approval was not required for this systematic review and meta-analysis as individual-level data were not used for this analysis.

#### Trial Inclusion Criteria

We included RCTs of parallel group design evaluating weight loss interventions administered for a minimum of 12 weeks in children and adolescents with overweight or obesity and younger than 18 years. The terms *overweight* (between 1 and 1.99 SD) and *obesity* (>2 SD for age and sex) were defined according to age- and sex-specific body mass index (BMI; calculated as weight in kilograms divided by height in meters squared) criteria for children and adolescents.^19,20^ We excluded trials evaluating pharmacotherapy for weight loss as well as cluster RCTs or quasi-experimental studies and those published in languages other than English. Cluster randomized trials were excluded as they would potentially increase trial heterogeneity, introduce difficulties in estimating intervention-type associations, and potentially be more common among layperson-led interventions, introducing a design bias into our analysis. RCTs that met inclusion criteria were classified into 3 comparisons: (1) professional-led vs standard, (2) layperson-led vs standard, and (3) professional-led vs layperson-led weight loss interventions.

#### Classification of Intervention Types

Professional-led interventions were defined as led by health care professionals, such as dieticians, nurses, kinesiologists, physicians, and other relevant certified health care professionals, at least twice during the conduct of the RCT. Direct involvement of laypeople (nonprofessionals) in participants’ schools, communities, neighborhoods, and families was considered a layperson-led weight loss intervention. Standard weight loss interventions (ie, standard of care) were defined as receiving recommendations for behavioral change without additional support provided to the participants at or before the baseline.

#### Study Selection

Abstracts and titles of relevant citations were independently screened by 2 of 6 reviewers (B.F.C., J.L., A.K.W., M.N.S., N.K., S.B.) to determine eligibility. Two reviewers independently assessed the eligibility of full-text articles of citations using a standardized prepiloted form outlining the inclusion and exclusion criteria. Disagreements were resolved by consensus or with the involvement of a third reviewer.

#### Data Extraction

Data were extracted independently by 2 of the 6 reviewers, with disagreements resolved by consensus or with the involvement of a third reviewer. For continuous data, we extracted change over time and the final reported measures of weight, as well as measures of variances, for each intervention type. We extracted outcome data from 2 time points—(1) immediately following the intervention and (2) at the end of the follow-up period—to assess long-term associations of the intervention. We used DistillerSR, version 2 (Evidence Partners Inc) to manage study selection, data extraction, and trial-level risk of bias assessments.

### Primary and Secondary Outcomes and Subgroup Analyses

The primary outcomes were the change from baseline in weight and any measure of BMI (BMI *z* score and BMI percentile) at the end of the intervention period. As outcomes for BMI were not consistently reported, we used the J correction factor for an unbiased estimate of the standardized mean difference (SMD).^21^ We also assessed change in percent body fat, waist circumference, and overall study withdrawals as secondary outcomes. For RCTs that reported long-term follow-up data after the end of the intervention, we also examined changes in these outcomes to assess the sustainability of the interventions. The change in the outcome variables was calculated as the difference between baseline and immediate postintervention measurements to calculate the short-term weight loss. The difference from baseline to the last follow-up after the intervention was completed was used to calculate the sustainability of the intervention.

### Risk of Bias Assessment

We evaluated the internal validity of included RCTs using the Cochrane risk of bias tool.^22^ This tool consists of 6 domains (sequence generation, allocation concealment, blinding, incomplete outcome data, selective outcome reporting, and other sources of bias) and a categorization of the overall risk of bias. Each separate domain was judged as low, unsure, or high risk of bias. The overall assessment was based on the responses to individual domains. If 1 or more individual domains were assessed as having a high risk of bias, the trial was judged as having a high risk of bias. The overall risk of bias was considered low only if no domain was rated as having an either high or unclear risk of bias. The source of funding was also extracted.

### Statistical Analysis

To rank the intervention types for relative effectiveness and to compare every intervention to each other using all available evidence, even when no studies contributed data directly, we used network meta-analyses (also termed as multiple, or mixed, treatment comparisons). We used a bayesian framework and Markov chain Monte Carlo simulation methods to combine direct and indirect evidence implemented in WinBUGS software, version 1.4.3 (University of Cambridge).^23^ We fit both random-effects and fixed-effect network meta-analysis models^24^ (code provided in eTable 2 of the Supplement). The preferred model was chosen by comparing the deviance information criteria^25^ (eTable 2 in the Supplement). For all analyses, we assessed model convergence using the Brooks-Gelman-Rubin diagnostic tool,^26^ history plots, autocorrelation, the form of the posterior density for the between-study heterogeneity, and the basic parameters (eFigures 1-4 in the Supplement). We used vague prior distributions for all parameters, a burn-in period of 50 000 iterations, a sampling period of 100 000 iterations, and 3 chains with varied initial values in all analyses (eTable 2 in the Supplement). The goodness of fit model was measured by the posterior mean of the residual deviance; in a well-fitting model, the residual deviance should be close to the number of data points included in the analysis.^27^ Where possible, we evaluated the consistency between the direct and indirect evidence by calculating a bayesian 2-sided *P* value for the difference between the direct and indirect estimates using the Bucher method,^28^ where the direct estimates were obtained from the inconsistency model (eTable 2 in the Supplement).^29,30,31^ *P* < .05 was considered significant.

Results are summarized by point estimates presented as medians with 95% CIs established using the 2.5 and 97.5 percentiles obtained via Markov chain Monte Carlo simulations. The 95% CI represents the interval in which the pooled effect is expected to lie with 95% probability. We also generated treatment rankings from best to worst and their corresponding probability estimates.

## Results

**Figure 1.**
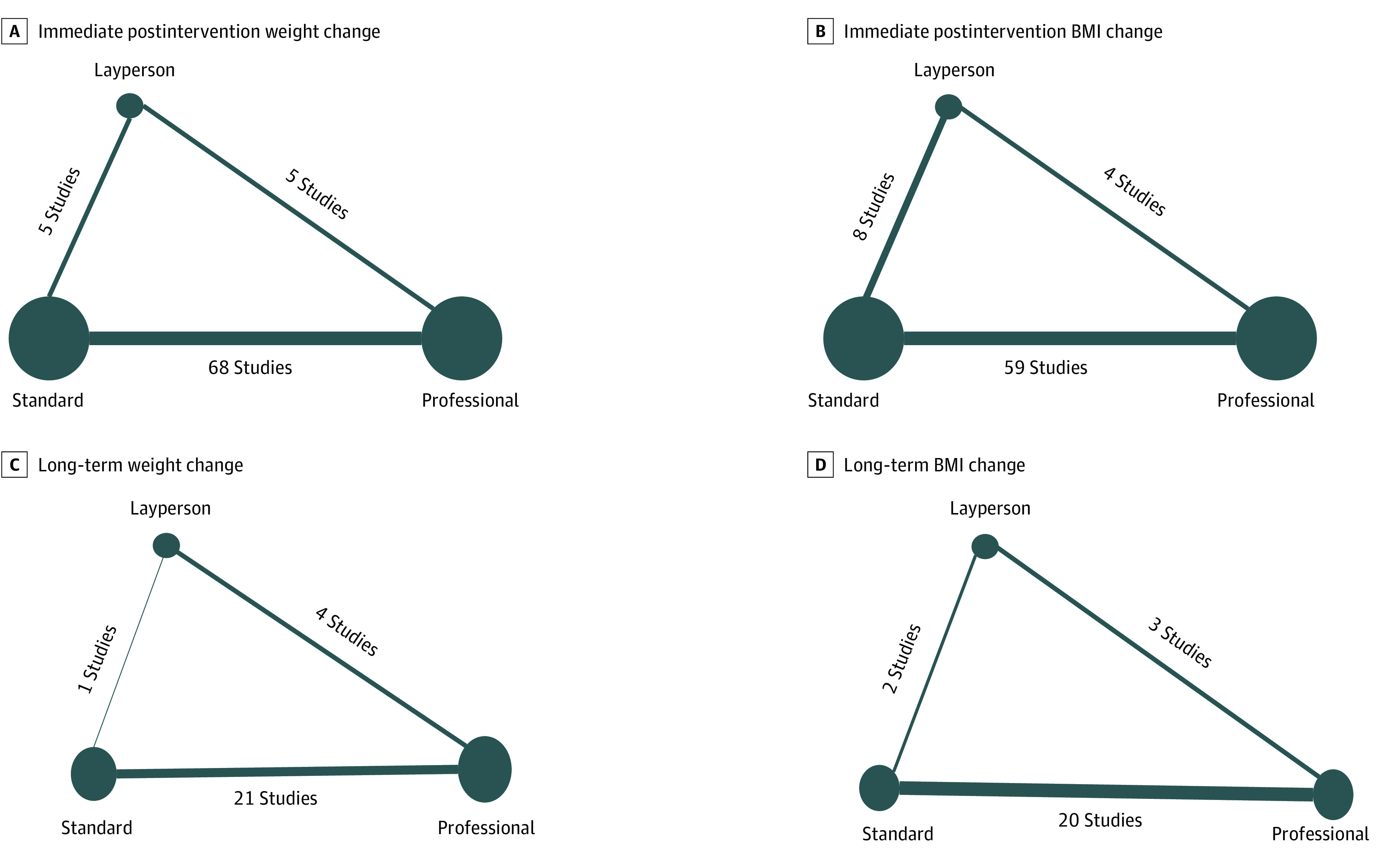
Network of Trials That Examined Layperson- and Professional-Led Approaches to Weight Loss Among Children and Adolescents With Overweight and Obesity Illustration of a network meta-analysis that combines direct evidence for the immediate postintervention change (A, B) and long-term change (C, D) in weight (A, C) and BMI (B, D) obtained from randomized clinical trials comparing 3 nodes: professional-led, layperson-led, and standard weight loss interventions. The size of the nodes is proportional to the number of participants randomized to that intervention type. The thickness of lines and the numbers represent the number of studies that contributed data for the comparison. Standard treatment considered as reference treatment for all network meta-analysis. BMI indicates body mass index.

We initially identified 25 586 citations and, after removing duplicates, we reviewed 20 514 unique citations. Of those, 78 RCTs^32,33,34,35,36,37,38,39,40,41,42,43,44,45,46,47,48,49,50,51,52,53,54,55,56,57,58,59,60,61,62,63,64,65,66,67,68,69,70,71,72,73,74,75,76,77,78,79,80,81,82,83,84,85,86,87,88,89,90,91,92,93,94,95,96,97,98,99,100,101,102,103,104,105,106,107,108,109,110,111,112,113,114,115^ (5780 participants) met the eligibility criteria (eFigure 1 in the Supplement). Details for each individual trial are presented in eTable 3 in the Supplement. The majority of RCTs were performed in children aged 1-12 years (n = 53)^9,11,32,33,39,40,43,44,45,46,48,49,52,53,54,56,58,59,62,63,64,67,68,69,70,71,73,76,77,80,83,84,85,86,87,88,89,91,92,93,95,99,100,101,102,103,104,107,108,110,111,112,114^; 25 were conducted in adolescents aged 13-18 years,^10,34,35,36,37,38,41,42,47,50,51,61,65,66,74,78,81,82,90,94,96,97,106,109,113,115,116,117,118^ and 3 studies did not report the mean age of participants.^72,98,106^ The proportion of male participants ranged from 0%-100%. Mean BMI percentile, BMI *z* score, and percent body fat were 96.9 (interquartile range [IQR], 90.2-99.2), 2.3 (IQR, 1.4-4.5), and 37.5% (IQR, 25.7%-47.6%), respectively. Only 25% of the RCTs were judged to have a low risk of bias (eTable 4 in the Supplement). The number of trials available for the 3 possible comparisons for immediate and long-term primary outcomes are presented in Figure 1. Across all trials, there was no evidence of inconsistency for any of the outcome measures included in the analyses.

### Intervention Details

Summary information for professional- and layperson-led interventions is provided in Table 1. The mean (SD) age (11.2 [3.5] vs 11.6 [3.9] years) and relative degree of obesity (mean [SD] BMI *z* score: 2.42 [0.57] vs 2.46 [0.31]) of participants that completed the trials was similar in professional- and layperson-led interventions. On average, each intervention type consisted of 1 to 1.5 hours of contact time, delivered 1 to 3 times per week for approximately 24 weeks.

**Table 1.** Behavioral Lifestyle Intervention Characteristics Between Layperson- and Professional-Led Trials for Children and Adolescents Living With Obesity

Intervention characteristic	Mean (SD)	
	Professional-led intervention	Layperson-led intervention
Trials, No.	78	5
Age, y	11.2 (3.5)	11.6 (3.9)
BMI *z* score	2.42 (0.57)	2.46 (0.31)
Contact time, h/wk^a^	1.6 (2.0)	1.2 (1.1)
Duration, wk	29 (22)	23 (7)

Abbreviation: BMI, body mass index.

^a^ Contact time is estimated time spent with person delivering the intervention each week during the intervention period.

### Primary Outcomes

**Figure 2.**
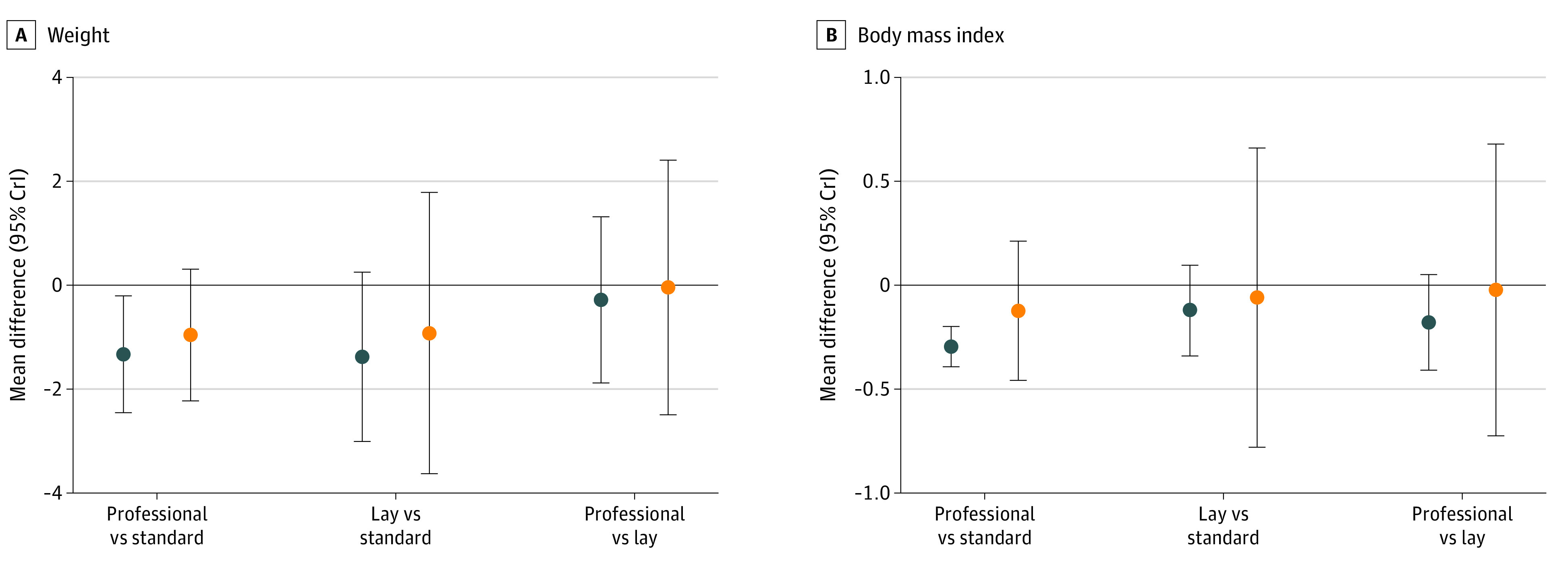
Short- and Long-term Efficacy of Layperson- and Professional-Led Weight Loss Interventions in Children and Adolescents With Overweight and Obesity A, Differences for weight outcomes are shown. B, Differences for body mass index are shown (standard mean difference). Results of the 3 possible comparisons within the network for the 2 primary outcome measures, body weight and body mass index, are displayed. From top to bottom in each panel, we compare professional-led interventions to standard of care, layperson-led interventions to standard of care, and head-to-head comparisons of professional- and layperson-led interventions. Blue circles represent immediate changes in outcomes; orange circles represent long-term (sustained) changes in outcomes. Horizontal lines reflect no difference between the intervention arm and the comparison arm. Whiskers indicate 95% CIs.

Data from each randomized trial on primary outcomes are presented in eFigure 2 in the Supplement. Random-effects models yielded better deviance information criteria than fixed-effects models for all models (eTable 2 in the Supplement). Total residual deviance and bayesian probability of inconsistency between direct and indirect effects in the models are presented in eTable 2 in the Supplement. Immediate and long-term changes in the 2 primary outcomes within the network meta-analysis are presented in Figure 2. The random-effects network meta-analysis revealed that professional-led behavioral interventions for children and adolescents were associated with a greater reduction in weight (mean difference [MD], –1.60 kg; 95% CI, –2.30 to –0.99 kg; *P* < .001) and BMI (SMD, –0.30; 95% CI, –0.39 to –0.20; *P* < .001) compared with standard care. Layperson-led weight loss interventions did not show an association with a difference in weight (MD, –1.40 kg; 95% CI, –3.00 to 0.26 kg; *P* = .05) or BMI (SMD, –0.12; 95% CI, –0.34 to 0.10; *P* = .14) compared with standard care. No differences were observed in RCTs that directly compared professional-led to layperson-interventions (weight MD, –0.25 kg; 95% CI, –1.90 to 1.30 kg; *P* = .38 and BMI SMD, –0.18; 95% CI, –0.41 to 0.05; *P* = .06).

For trials with prolonged follow-up, neither professional-led interventions (MD, –1.02 kg; 95% CI, –2.20 to 0.34 kg; *P* = .06) or layperson-led interventions (MD, –0.98 kg; 95% CI, –3.60 to 1.80 kg; *P* = .23) were associated with reduction in weight following discontinuation of the intervention, compared with standard care (Figure 2).

### Secondary Outcomes

Results for associations between layperson- and professional-led behavioral interventions and secondary outcomes are presented in Table 2. Professional-led interventions were associated with significant reductions in percent body fat (MD, –1.70%; 95% CI, –2.60% to –0.81%; *P* < .001) and waist circumference (MD, –1.30 cm; 95% CI, –2.06 to –0.58 cm; *P* < .001) compared with standard care. No differences in percent body fat (MD, –0.52%; 95% CI, –3.90% to 2.80%; *P* = .38) or waist circumference (MD, –0.94 cm; 95% CI, –2.70 to 0.71 cm; *P* = .13) following layperson-led interventions were seen compared with standard care (Table 2). There were insufficient data to analyze long-term associations of secondary outcomes. No differences were observed in either professional- or layperson-led interventions for study withdrawals.

**Table 2.** Secondary Outcomes of Layperson- and Professional-Led Weight Loss Interventions for Children and Adolescents With Overweight and Obesity for Body Composition and Study Withdrawals

Outcome	No.^a^	Professional vs standard, mean difference (95% CI)	No.^a^	Layperson vs standard, mean difference (95% CI)	No.^a^	Professional vs layperson, mean difference (95% CI)
Body fat, %	36	−1.70 (−2.60 to −0.81)	0	−0.52 (−3.90 to 2.80)	2	−1.13 (−4.40 to 2.20)
Waist circumference, cm	33	1.30 (−2.06 to −0.58)	5	−0.94 (−2.70 to 0.71)	1	−0.34 (−2.15 to 1.50)
BMI, %	12	−0.59 (−1.45 to 0.23)	0	0.01 (−1.93 to 2.05)	2	−0.59 (−2.49 to 1.14)
Study withdrawals, OR (95% CI)	65	0.92 (0.78 to 1.11)	7	0.99 (0.63 to 1.58)	5	0.93 (0.59 to 1.47)

Abbreviations: BMI, body mass index; OR, odds ratio.

^a^ Indicates the number of trials included in the analysis.

### Treatment Rankings

**Figure 3.**
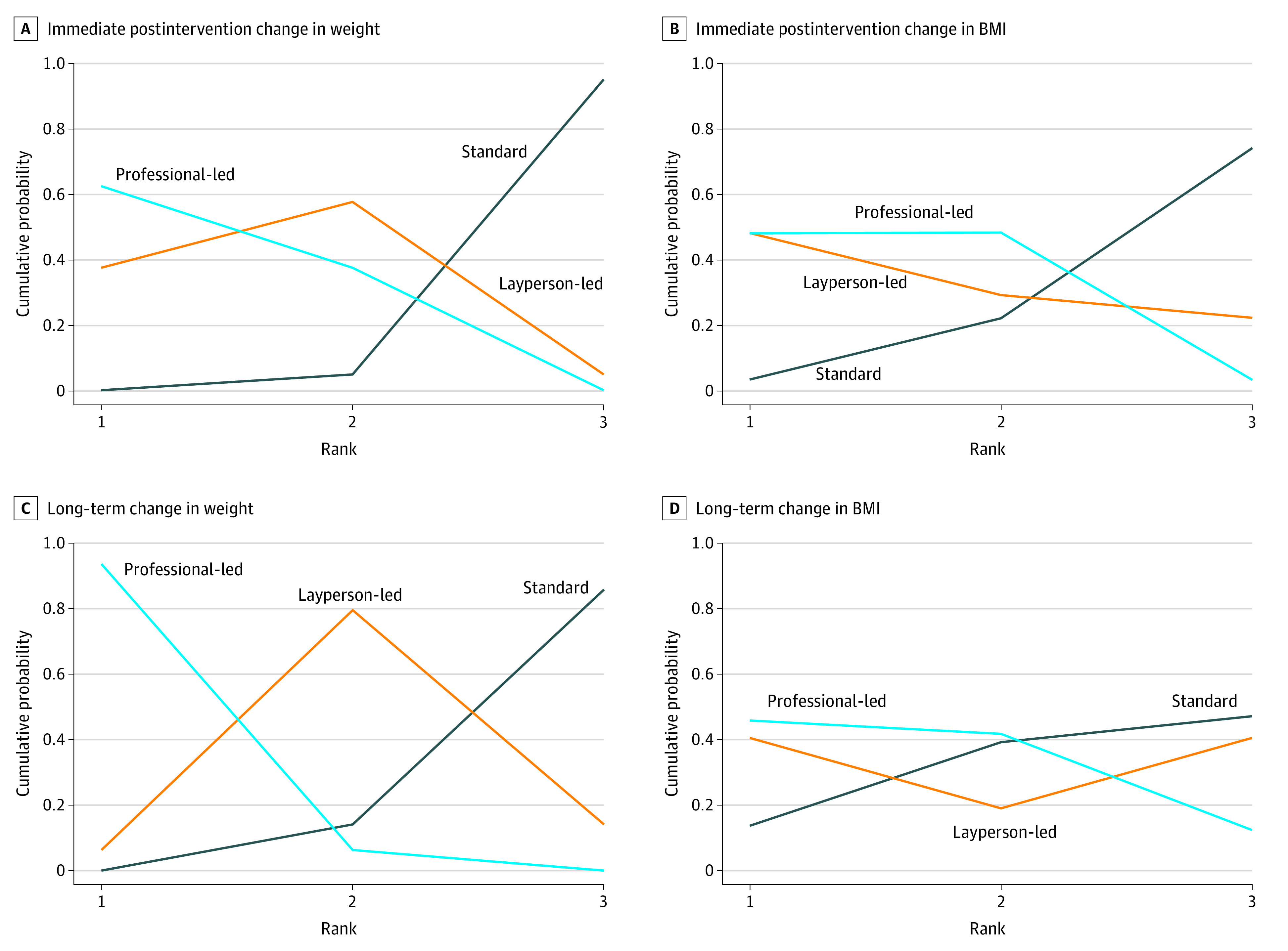
Ranked Intervention Types for Short- and Long-term Weight Loss Among Children and Adolescents With Overweight and Obesity Surface under the cumulative ranking curve–based treatment rankings for immediate postintervention change (A, B) and long-term change (C, D) in weight (A, C) and BMI (B, D) obtained from randomized clinical trials. Data represent the probability of being ranked as the best (1), next best (2), and least effective (3) intervention. BMI indicates body mass index.

Treatment rankings for the 3 intervention types for both primary outcomes, immediately following the intervention and during long-term follow-up, are presented in Figure 3. The cumulative probabilities of each treatment are presented in eTables 4 and 5 in the Supplement. For both primary outcomes, professional-led interventions were considered the best approach to achieve short-term absolute (mean [SD] rank, 1.38 [0.48]) and relative (mean [SD] rank, 1.06 [0.24]) weight reduction (Figure 3). Layperson-led interventions were considered the second-best intervention for absolute (mean [SD] rank, 1.67 [0.56]) weight loss and relative (mean [SD] rank, 2.08 [0.45]) weight loss immediately following the intervention. For long-term follow-up, professional- and layperson-led interventions were ranked nearly equal for their association with achieving absolute and relative weight loss (Figure 3).

## Discussion

The main finding from this systematic review and network meta-analysis was that professional-led behavioral weight loss interventions were associated with significant short-term, but not long-term, reductions in weight and BMI in children and adolescents with overweight or obesity. There was no association between layperson-led behavioral weight loss interventions and weight reduction among children and adolescents with overweight or obesity. In the absence of direct evidence and low precision for the indirect evidence, it is unclear how layperson-led interventions compare with professional-led interventions for achieving weight loss among children and adolescents with overweight or obesity. Finally, the degree of weight loss achieved with behavioral lifestyle interventions was modest (–1.0 to –2.3 kg) regardless of intervention type.

In 2017, the US Preventive Services Task Force released the results from an extensive systematic review of trials examining weight loss interventions for children and adolescents with overweight or obesity.^4^ This review tested for differences in treatment outcomes across studies that had different contact time with participants, but did not directly compare different intervention models. Among the 42 behavioral therapeutic trials included in the analysis, several were not randomized, cluster trials were included, and no long-term follow-up data were provided. Despite these differences between the network meta-analysis presented here and the analysis from the US Preventive Services Task Force, the effect size for professional-led interventions was similar and comparable to previous systematic reviews of weight loss interventions among children and adolescents with overweight or obesity.^3,116,117^ The data presented here extend previous systematic reviews by demonstrating that the short-term benefits of professional-led weight loss interventions are not sustained following the end of the intervention and that similar effect sizes may be achieved with layperson-led interventions. Adherence to lifestyle change is a critical determinant of intervention effectiveness. Unfortunately, very few trials reported adherence to prescribed lifestyle change, and therefore it is unclear whether the lack of maintenance and the relatively modest weight loss following these interventions are associated with low adherence to intervention attributes. Collectively, these data provide robust evidence that professional-led behavioral interventions are associated with achieving modest weight loss among children and adolescents with overweight or obesity; however, this association was not sustained in the long term.

Systematic reviews of home-,^119^ school-,^118^ and community-based^120^ behavioral interventions for obesity prevention in children and adolescents suggest that these nonprofessional-led interventions yield minimal or no weight change. In contrast to previous systematic reviews, we excluded cluster randomized trials, quasi-experimental trials, trials lasting less than 12 weeks, and trials that included children and adolescents of a healthy weight. For the current review, layperson-led interventions were delivered either by parents or older peers without formal training in a health profession. In contrast to results from quasiexperimental^6,7,121^ or cluster randomized trials,^8^ the RCTs of layperson-led interventions examined here did not show an association in the short or long term. The effect sizes for absolute (–1.60 vs –1.40 kg) and relative (BMI, –0.3 vs –0.12) weight loss were similar between layperson- and professional-led interventions. The few trials of layperson-led interventions, however, lacked precision. Larger trials of layperson-led interventions, particularly trials directly comparing layperson- and professional-led interventions, are needed to understand the association of this approach for weight management among children and adolescents with overweight or obesity.

There is some evidence that layperson- or peer-led approaches support positive behavioral change and improved health outcomes among adults living with obesity^122,123,124,125^ or obesity-related comorbidities.^126^ Layperson- or community-led interventions have proved to be associated with low-resource areas or settings in which culturally tailored approaches are preferred by community members.^12,13^ The meta-analysis conducted here found that layperson-led behavioral trials were not associated with statistically significant reductions in body weight among children and adolescents living with obesity. Weight status is only 1 of multiple measures of health that can be influenced by behavioral change in children and adolescents with overweight or obesity, particularly cardiometabolic risk factors. Children and adolescents with overweight or obesity also are more likely to live in families with low income,^127,128^ to have been exposed to adverse childhood experiences, and to suffer from mental health comorbidities. We did not include these outcomes in our analysis; however, it is possible that these outcomes could be responsive to layperson-led interventions. The promising association of layperson-led approaches in other settings and populations^129,130^ reinforces the need for large-scale, well-designed, multiarm RCTs to determine the effectiveness of interventions led by lay individuals for supporting weight change among children and adolescents with overweight or obesity.

The advantage of conducting a network meta-analysis, relative to a conventional meta-analysis, lies in the capacity to estimate the relative efficacy of 2 given interventions when few or no direct head-to-head trials exist. We were only able to identify 3 to 5 trials that directly compared layperson- to professional-led interventions for weight loss in children and adolescents with overweight or obesity. Performing a meta-analysis on the results of these RCTs did not reveal an association with either intervention. The few RCTs directly comparing layperson- and professional-led interventions were relatively low-powered and were considered to have a high risk of bias. Based on the limited available evidence, professional-led approaches were ranked as being associated with short-term weight loss; however, over the long-term, layperson- and professional-led interventions appeared to perform equally. Adequately powered, head-to-head trials of layperson- and professional-led approaches with long-term follow-up are needed to confirm these observations.

### Strengths and Limitations

The study is strengthened by limiting the analyses to trials focused exclusively on children and adolescents with overweight or obesity, an a priori published protocol, and the relatively large number of RCTs available for the network meta-analysis. Despite these strengths, there are limitations to consider. The strict criteria we imposed on the search limited the inclusion of designs, including cluster RCTs and quasi experiments, which limits the generalizability of our findings. Additionally, differences in intervention designs could have influenced the point estimates between professional- and layperson-led trials; however, with only 5 trials led by laypersons, we were underpowered to adjust for these differences. We also recognize that age- and sex-standardized measures of adiposity are the best practice for reporting weight-related outcomes in children. As we relied on published outcome data and not individual-level data, we were largely unable to use BMI or waist circumference *z* scores. Additionally, we only searched for trials appearing in the last 20 years in an effort to limit the number of low-quality RCTs. We did not include non-English publications or RCTs that were unpublished in order to increase feasibility and the homogeneity between weight loss interventions; this may have introduced selective reporting bias (eg, publication bias). Furthermore, only 25% of the included trials were judged as having a low risk of bias. As these were behavioral trials, blinding was not possible; however, as the outcomes are semiobjective, blinding may not be as effective as in pharmaceutical trials with subjective outcomes.^131^ As mentioned previously, this review was restricted to weight-related outcomes and did not include other outcomes that could be responsive to behavioral lifestyle change. Finally, there were very few trials that directly compared the effectiveness of layperson- and professional-led interventions. This limits our ability to provide an accurate estimate of the effects and also resulted in very low precision. Similarly, with so few layperson-led interventions with long-term follow-up, the precision was very low for the indirect comparisons generated by the network meta-analysis.

## Conclusions

In this systematic review and meta-analysis, professional-led behavioral interventions were associated with modest but statistically significant weight loss among children and adolescents with overweight or obesity, compared with standard weight loss interventions. Layperson-led behavioral interventions were no associated with weight loss. Weight loss achieved by both professional and layperson-led interventions were not sustained following the intervention among children and adolescents with overweight or obesity. These findings suggest a need for trials assessing the immediate and sustained effectiveness of layperson-led behavioral weight loss interventions among children and adolescents with overweight or obesity.

## Notes

Supplement. **eFigure 1.** PRISMA Flow Diagram for Trials That Were Included in the Analyses **eFigure 2.** Individual Results From Randomized Trials of Weight Loss Interventions Delivered by Professionals or Lay Persons on Weight Loss Among Overweight and Obese Children and Adolescents **eFigure 3.** Bayesian Model Inference Data for Weight Change Immediately Following the Intervention **eFigure 4.** Treatment Rankings for Intervention Types for Immediate and Long-term Weight Change **eTable 1.** Search Strategy for Medline **eTable 2.** Additional Statistical Methods **eTable 3.** Baseline Characteristics of Included Randomized Clinical Trials **eTable 4.** Cochrane Risk of Bias Assessment for Eligible RCTs **eTable 5.** Mean and Median Probabilities of Treatment Ranks
