# Supplementary material for: Layperson-Led vs Professional-Led Behavioral Interventions for Weight Loss in Pediatric Obesity: A Systematic Review and Meta-analysis
Source: JAMA Netw Open. 2020 Jul 13;3(7):e2010364. doi: 10.1001/jamanetworkopen.2020.10364 (PMC7358915; doi:10.1001/jamanetworkopen.2020.10364)
Supplement: Supplement. — eFigure 1. PRISMA Flow Diagram for Trials That Were Included in the Analyses eFigure 2. Individual Results From Randomized Trials of Weight Loss Interventions Delivered by Professionals or Lay Persons on Weight Loss Among Overweight and Obese Children and Adolescents eFigure 3. Bayesian Model Inference Data for Weight Change Immediately Following the Intervention eFigure 4. Treatment Rankings for Intervention Types for Immediate and Long-term Weight Change eTable 1. Search Strategy for Medline eTable 2. Additional Statistical Methods eTable 3. Baseline Characteristics of Included Randomized Clinical Trials eTable 4. Cochrane Risk of Bias Assessment for Eligible RCTs eTable 5. Mean and Median Probabilities of Treatment Ranks [file jamanetwopen-3-e2010364-s001.pdf]

Supplementary Online Content

McGavock J, Chauhan BF, Rabbani R, et al. Layperson-led vs professional-led behavioral interventions for weight loss in pediatric obesity: a systematic review and meta-analysis. *JAMA Netw Open*. 2020;3(7):e2010364.
doi:10.1001/jamanetworkopen.2020.10364

eFigure 1. PRISMA Flow Diagram for Trials That Were Included in the Analyses

eFigure 2. Individual Results From Randomized Trials of Weight Loss Interventions Delivered by Professionals or Lay Persons on Weight Loss Among Overweight and Obese Children and Adolescents

eFigure 3. Bayesian Model Inference Data for Weight Change Immediately Following the Intervention

eFigure 4. Treatment Rankings for Intervention Types for Immediate and Long-term Weight Change

eTable 1. Search Strategy for Medline

eTable 2. Additional Statistical Methods

eTable 3. Baseline Characteristics of Included Randomized Controlled Trials

eTable 4. Cochrane Risk of Bias Assessment for Eligible RCTs

eTable 5. Mean and Median Probabilities of Treatment Ranks

This supplementary material has been provided by the authors to give readers additional information about their work.

© 2020 McGavock J et al. *JAMA Network Open.*

eFigure 1. PRISMA Flow diagram for trials that were included in the analyses.

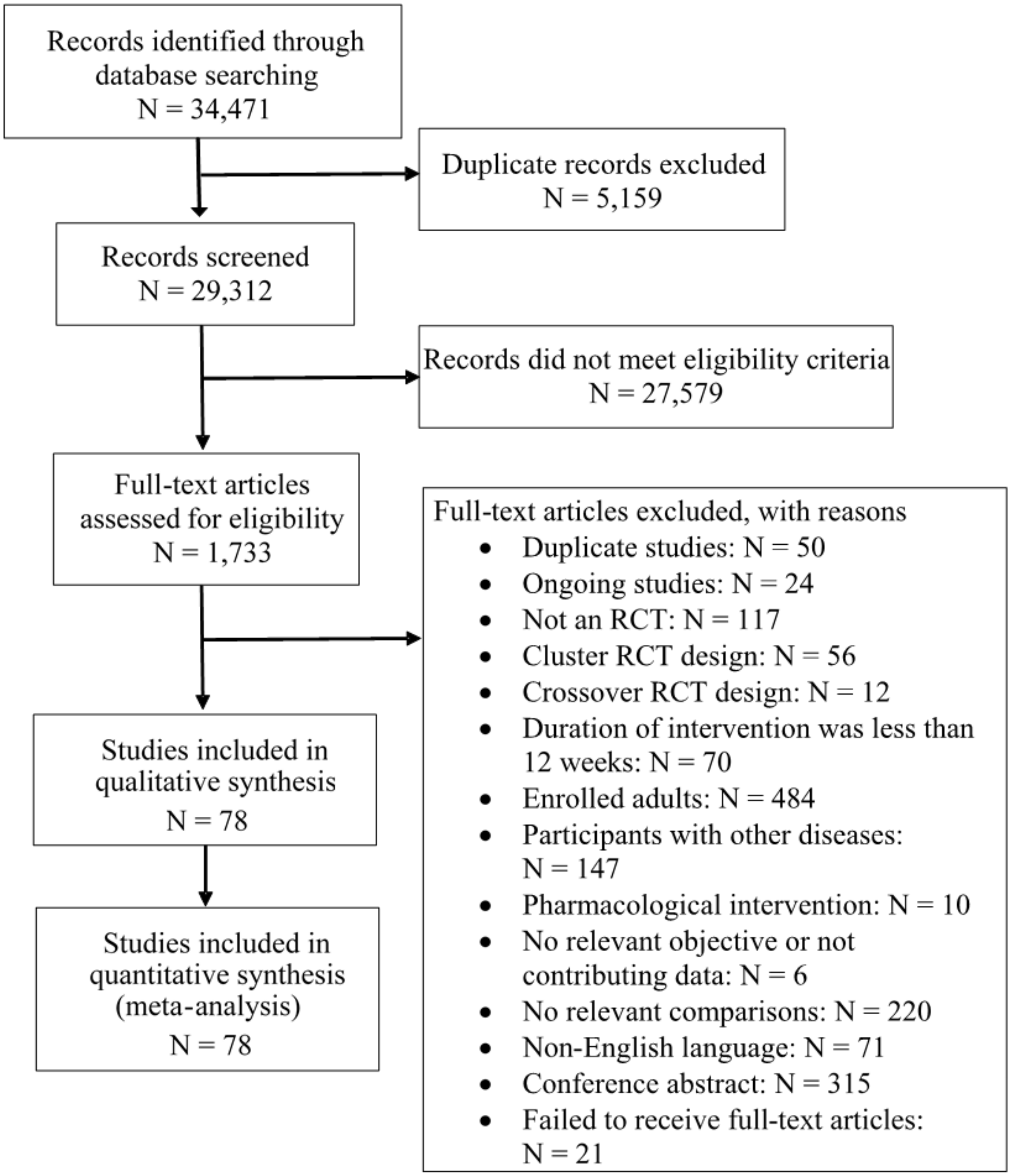

© 2020 McGavock J et al. *JAMA Network Open.*

eFigure 2. Individual results from randomized trials of weight loss interventions delivered by professionals or laypersons on weight loss among overweight and obese children and adolescents.

A - Weight

	Experimental			Comparator			Mean Difference
Study or Subgroup	Mean	SD	Total	Mean	SD	Total	IV, Random, 95% CI
**1.1.1 Professional vs Standard**							
Balagopal 2005	104.5	34.13019	7	117.3	14.99066	8	-12.80 [-40.13, 14.53]
Ball 2011	97.2	16.07599	11	109.9	18.17275	10	-12.70 [-27.43, 2.03]
Ball_a 2011	103.3	16.07599	11	109.9	19.38418	9	-6.60 [-22.43, 9.23]
Berkowitz 2013	0.61	7.571314	53	0.4	7.654045	61	0.21 [-2.59, 3.01]
Berntsen 2010	75	12.19827	16	76	13.86815	32	-1.00 [-8.67, 6.67]
Bocca 2014	-0.198	1.4214	29	0.391	1.3825	33	-0.59 [-1.29, 0.11]
Bocca_a 2012	-0.2	1.4	29	0.4	1.4	33	-0.60 [-1.30, 0.10]
Boff 2018	96.77	20.73	34	94	16.28	34	2.77 [-6.09, 11.63]
Butte_a 2017	24.7	4.64758	60	22.9	5	100	1.80 [0.27, 3.33]
Butte_b 2017	40.7	8.246211	68	40.4	8.504117	113	0.30 [-2.21, 2.81]
Butte_c 2017	57	11.32519	106	60.5	11.10946	102	-3.50 [-6.55, -0.45]
Chae 2010	-2.3	10.02547	19	2.5	9.153688	19	-4.80 [-10.90, 1.30]
Chae_a 2010	-2.3	10.02547	19	2.5	9.153688	19	-4.80 [-10.90, 1.30]
Christie 2017	2	21.45297	87	1.77	19.5875	87	0.23 [-5.87, 6.33]
Crabtree 2010	72.7	16.1	9	74.1	17.5	10	-1.40 [-16.51, 13.71]
Cvetkovic_a 2018	62.81	15.28	14	71.18	13.06	10	-8.37 [-19.75, 3.01]
Cvetkovic_b 2018	71.53	15.28	14	71.18	11.23	11	0.35 [-10.05, 10.75]
Cvetkovic_c 2018	-0.89	6.693999	14	3.83	2.02696	10	-4.72 [-8.44, -1.00]
Cvetkovic_d 2018	0.04	6.693999	14	3.83	2.292318	11	-3.79 [-7.55, -0.03]
Debar 2012	86.21	16.93	103	86.19	16.08	105	0.02 [-4.47, 4.51]
Dias 2018	72.8	20.5	34	72.8	20.6	32	0.00 [-9.92, 9.92]
Dias_a 2018	70.7	20.5	34	72.8	12.9	33	-2.10 [-10.28, 6.08]
Diaz 2010	-0.8	5.9	22	5.6	5.2	21	-6.40 [-9.72, -3.08]
Diaz_a 2010	69.5	15.9	33	74.8	16.1	33	-5.30 [-13.02, 2.42]
Doyle 2008	97.23	21.41	40	95.6	29.13	40	1.63 [-9.57, 12.83]
Epstein 2000	51.2	10.8	17	50.8	11.3	18	0.40 [-6.92, 7.72]
Epstein_a 2000	57.4	10.8	17	50.8	11.8	17	6.60 [-1.00, 14.20]
Faude 2010	69.5	14.2	11	68.6	12.3	11	0.90 [-10.20, 12.00]
Hay 2016	89.9	18.6	36	95	16.02748	38	-5.10 [-13.03, 2.83]
Hay_a 2016	87.2	18.6	36	95	16.40488	32	-7.80 [-16.12, 0.52]
Hofsteenge 2014	95.9	18.8	51	95.1	17.7	71	0.80 [-5.80, 7.40]
Hughes 2008	54.6	17.7	48	54.2	12.89995	49	0.40 [-5.77, 6.57]
Kalarchian 2009	1.56	5.555673	95	4.76	6.697223	97	-3.20 [-4.94, -1.46]
Kim 2011	80.7	10.66	12	87.84	13.19	18	-7.14 [-15.71, 1.43]
Kong 2014	84.3	13.1	52	83	14.4	52	1.30 [-3.99, 6.59]
Leite 2013	75.7	20.7	16	75.02	14.6	43	0.68 [-10.36, 11.72]
Lison_a 2012	75.2	63.19684	24	77	43.335	45	-1.80 [-30.08, 26.48]
Nemet_a 2008	55.9	17.24645	11	57.6	15.9198	11	-1.70 [-15.57, 12.17]
Nemet_b 2005	61	24.1	22	64.5	18.3	24	-3.50 [-15.95, 8.95]
Nobre 2016	42.7	11.33314	19	44.9	6.957011	40	-2.20 [-7.73, 3.33]
Pedrosa 2011	43.9	8	42	46.5	6.4	19	-2.60 [-6.36, 1.16]
Quattrin 2014	25.1	2.12132	50	26.4	2.034699	46	-1.30 [-2.13, -0.47]
Racil_a 2016	80.7	4.9	19	81.3	5.7	23	-0.60 [-3.81, 2.61]
Racil_b 2016	80.8	4.9	19	81.3	5	26	-0.50 [-3.42, 2.42]
Racil_c 2016	84.1	4.2	14	85.9	4.7	17	-1.80 [-4.94, 1.34]
Racil_d 2016	86.6	4.2	14	85.9	6.5	16	0.70 [-3.17, 4.57]
Rebai 2013	59.2	11.9	14	55.8	9.1	14	3.40 [-4.45, 11.25]
Saelens 2002	86.1	13.8	21	84.1	14	23	2.00 [-6.22, 10.22]
Savoye_a 2014	0.6	3.91823	27	3.7	4.089389	31	-3.10 [-5.16, -1.04]
Savoye_b 2007	0.3	9.782446	69	7.7	8.784411	105	-7.40 [-10.25, -4.55]
Schwingshandl 1999	62.6	19.7	16	68.7	14.8	14	-6.10 [-18.48, 6.28]
Shaibi 2006	91.9	21.55806	11	100	18.5731	11	-8.10 [-24.92, 8.72]
Sigal_a 2014	97	16.56382	76	99.2	16.45448	75	-2.20 [-7.47, 3.07]
Sigal_b 2014	100.4	16.56382	76	99.2	16.78035	78	1.20 [-4.07, 6.47]
Sigal_c 2014	97	16.56382	76	99.2	16.45448	75	-2.20 [-7.47, 3.07]
Sigal_d 2014	-0.1	7.220697	76	1.3	6.95413	75	-1.40 [-3.66, 0.86]
Sigal_e 2014	0.3	7.220697	76	1.3	7.09644	78	-1.00 [-3.26, 1.26]
Sigal_f 2014	-0.8	7.220697	76	1.3	6.736813	75	-2.10 [-4.33, 0.13]
Soltero 2018	95.1	19.9359	69	97.9	18.82631	67	-2.80 [-9.32, 3.72]
Stark_a 2014	-0.1	0.9	12	1.9	2.3	11	-2.00 [-3.45, -0.55]
Stark_b 2014	1.1	0.9	12	1.9	2.4	10	-0.80 [-2.37, 0.77]
Stark_c 2011	-0.9	0.9	10	1.8	2.3	7	-2.70 [-4.49, -0.91]
Taylor 2015	37.9	8.8	102	35.5	11.5	104	2.40 [-0.39, 5.19]
Wesnigk_a 2016	75	22.6	8	117.2	10.7	8	-42.20 [-59.53, -24.87]
Wesnigk_b 2016	1.07	0.8	8	2.93	0.67	8	-1.86 [-2.58, -1.14]
Williamson 2006	4.4	8.616264	29	6.3	8.995554	28	-1.90 [-6.48, 2.68]
Yusop 2018	57.3	11.2	20	59.5	11.4	20	-2.20 [-9.20, 4.80]
Zehsaz 2016	54.5	3.5	16	59.5	3.4	16	-5.00 [-7.39, -2.61]
**1.1.2 Lay vs Standard**							
Foster 2016	27.76	5.559866	25	25.63	7.295938	23	2.13 [-1.56, 5.82]
Kulik 2015	-2.9	3.22	17	-2.93	3.76	19	0.03 [-2.25, 2.31]
Lison_c 2012	66.9	63.19684	24	77	30.09468	41	-10.10 [-37.01, 16.81]
Wengle_a 2011	88.7	5.5	16	90.6	18.8	16	-1.90 [-11.50, 7.70]
Wengle_b 2011	88.7	5.5	16	90.6	18.8	16	-1.90 [-11.50, 7.70]
**1.1.3 Professional vs Lay**							
Arlinghaus 2017	2.03	3.82	95	1.18	2.89	94	0.85 [-0.12, 1.82]
Jelalian 2006	-3.2	3.61	39	-5.31	5.61	37	2.11 [-0.02, 4.24]
Jelalian_a 2010	82.41	14.66	56	81.52	13.87	62	0.89 [-4.27, 6.05]
Johnston 2007	1.9	2.7	46	3.49	2.74	25	-1.59 [-2.92, -0.26]
Lison_b 2012	75.2	43.335	45	66.9	30.09468	41	8.30 [-7.36, 23.96]

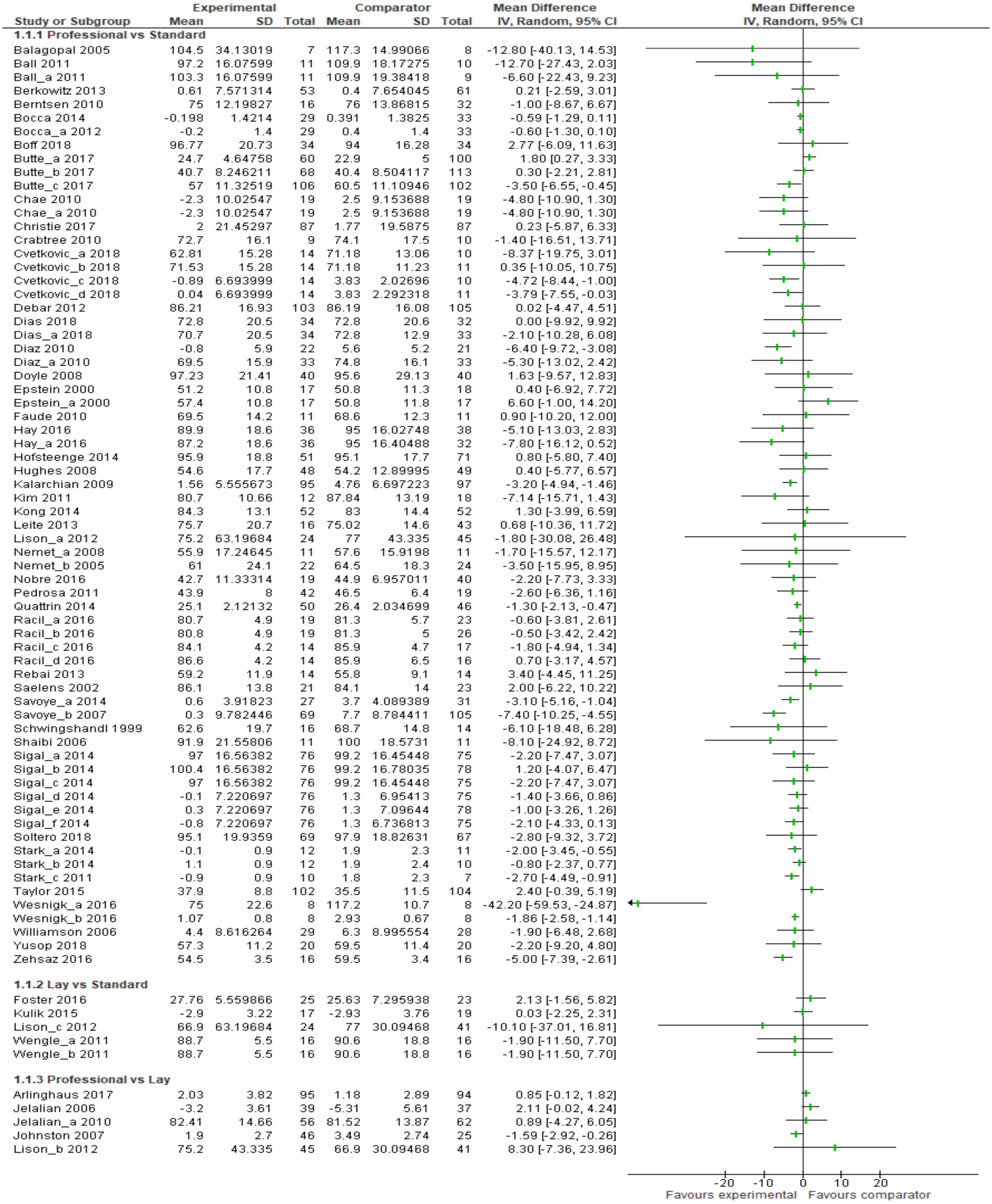

© 2020 McGavock J et al. *JAMA Network Open.*

B. Body Mass Index

Study or Subgroup	Treatment Mean	Treatment SD	Treatment Total	Comparator Mean	Comparator SD	Comparator Total	Std. Mean Difference IV, Random, 95% CI
**1.1.1 Professional vs Standard**							
Abraham 2015	-0.015	3.29837899	16	0.31	3.93632662	16	-0.09 [-0.78, 0.61]
Anderson 2017	-0.4	0.44977772	56	-0.38	0.55865911	57	-0.04 [-0.41, 0.33]
Arlinghaus 2017	-0.14	2.638257	69	-0.09	2.65135814	69	-0.02 [-0.35, 0.31]
Ball 2011	-0.91	4.8	19	0.8	4.99	22	-0.34 [-0.96, 0.28]
Berkowitz 2013	-0.45	2.73358739	61	-0.38	2.62083956	53	-0.03 [-0.39, 0.34]
Bocca 2014	-1.154	0.968	33	-0.614	1.112	29	-0.51 [-1.02, -0.01]
Bocca_a 2012	-1.2	1	33	-0.6	1.1	29	-0.57 [-1.07, -0.06]
Boff 2018	-0.78	4.33055424	34	-0.5	5.74511967	34	-0.05 [-0.53, 0.42]
Broccoli 2016	0.46	1.34712193	186	0.78	1.28467418	185	-0.24 [-0.45, -0.04]
Butte 2017	-0.7	3.70405184	33	-0.9	4.51331364	34	0.05 [-0.43, 0.53]
Chae 2010	-1.6	3.29089653	19	0.5	4.592385	19	-0.51 [-1.16, 0.13]
Christie 2017	-0.2	3	100	-0.1	2.79284801	60	-0.03 [-0.35, 0.29]
Crabtree 2010	-0.3	5.21152569	10	-0.1	3.21869539	9	-0.04 [-0.94, 0.86]
Cvetkovic 2018	-0.78	4.13762009	10	0.87	4.63634554	14	-0.36 [-1.18, 0.46]
Davis 2013	-0.12	0.52	20	-0.15	0.53394756	22	0.06 [-0.55, 0.66]
Debar 2012	-0.12	0.3798684	105	-0.06	0.3576311	103	-0.16 [-0.43, 0.11]
Diaz 2010	-1.8	5.45068803	33	0.4	4.47101778	33	-0.44 [-0.92, 0.05]
Doyle 2008	-0.08	0.50507425	40	0.01	0.4350862	40	-0.19 [-0.63, 0.25]
Epstein 2000	-0.6	3.15119025	14	0.5	3.8509739	14	-0.30 [-1.05, 0.44]
Faude 2010	0.5	3.11929479	11	0.6	3.83536178	11	-0.03 [-0.86, 0.81]
Foster 2016	-2.1	1.2489996	16	0.2	1	16	-1.98 [-2.85, -1.12]
Gerards 2015	-0.27	1.31476788	45	0.07	1.3301305	46	-0.25 [-0.67, 0.16]
Golley 2007	-1.3	0.9	17	-1.5	0.8	17	0.23 [-0.45, 0.90]
Hofsteenge 2014	-0.5	4.65080638	71	0.6	5.20288382	51	-0.22 [-0.58, 0.14]
Hughes 2008	-0.21	0.69	49	-0.1	0.61	48	-0.17 [-0.57, 0.23]
Kalarchian 2009	-0.68	2.85616876	97	0.54	2.04682681	95	-0.49 [-0.78, -0.20]
Kelishadi 2008	-1.1	0.3	47	-1.04	0.2	45	-0.23 [-0.64, 0.18]
Kim 2011	0.3	5.30659966	11	0.4	6.63324958	11	-0.02 [-0.85, 0.82]
Kokkvoll 2015	1.29	1.83068945	45	2.02	1.95310301	46	-0.38 [-0.80, 0.03]
Kong 2014	-1.2	4.10365691	52	-0.2	3.24191302	52	-0.27 [-0.65, 0.12]
Leite 2013	-0.7	4	43	-0.11	6.2	16	-0.12 [-0.70, 0.45]
Lison 2012	-0.4	7.72124342	45	1.6	7.66647898	24	-0.26 [-0.75, 0.24]
Nemet 2008	-0.7	6.14247507	11	-0.1	4.48664686	11	-0.11 [-0.94, 0.73]
Nemet_a 2005	-1.7	4.00374824	24	-0.2	5.32541078	22	-0.31 [-0.90, 0.27]
Nobre 2016	-11.98	5.38615819	8	0.22	6.06094877	8	-2.01 [-3.28, -0.74]
Norman 2016	0.22	4.02	53	0.41	3.92	53	-0.05 [-0.43, 0.33]
Pedrosa 2011	-0.16	1.7027331	19	0.16	2.65384626	42	-0.13 [-0.67, 0.41]
Quattrin 2014	-0.45	0.3391165	46	-0.21	0.35355339	50	-0.69 [-1.10, -0.27]
Racil 2016	-0.3	0.36055513	17	0	0.45825757	14	-0.72 [-1.45, 0.02]
Rebai 2013	-0.49	0.36	7	0.1	0.32	10	-1.66 [-2.82, -0.51]
Saelens 2002	-0.1	3.65923489	23	1.1	3.26036808	21	-0.34 [-0.94, 0.26]
Sauder 2018	0.18	10.73	87	0.25	3.5	87	-0.01 [-0.31, 0.29]
Savoye 2014	-0.37	1.02234725	31	0.67	1.36506063	27	-0.86 [-1.40, -0.32]
Savoye_a 2007	-1.7	3.10038029	105	1.6	3.12205708	69	-1.06 [-1.38, -0.73]
Schwingshandl 1999	4.78654706	4.01371143	14	4.78138046	4.13620484	16	0.00 [-0.72, 0.72]
Seo 2019	-0.08	0.54110997	26	-0.03	0.50685304	44	-0.10 [-0.58, 0.39]
Serra-Paya 2015	-0.12	0.22	54	-0.09	0.23	59	-0.13 [-0.50, 0.24]
Sigal 2014	-0.5	0.26457513	23	-0.1	0.3	19	-1.40 [-2.08, -0.71]
Soltero 2018	-0.8	5.36749476	67	0.3	5.8146367	69	-0.20 [-0.53, 0.14]
Stark 2011	-1.52	3.74669187	18	0.15	2.87047034	12	-0.47 [-1.22, 0.27]
Stovitz 2014	-0.04	0.35496479	35	-0.07	0.36	36	0.08 [-0.38, 0.55]
Taylor 2015	0.8	2.9816103	104	1.2	2.29128785	102	-0.15 [-0.42, 0.12]
Wake 2013	0.85	3.38926246	56	0.84	4.19986905	49	0.00 [-0.38, 0.39]
Weigel 2008	-1.5	3.04466747	36	2.8	3.85875628	30	-1.24 [-1.77, -0.71]
Wengle 2011	-0.5	4.82182538	75	0	4.85386444	76	-0.10 [-0.42, 0.22]
Wilfley 2017	-0.05	0.13665902	61	-0.03	0.19148182	49	-0.12 [-0.50, 0.25]
Williamson 2006	0.73	3.49239173	28	1.2	3.50035712	29	-0.13 [-0.65, 0.39]
Yusop 2018	-0.2	0.55677644	20	-0.1	0.8	20	-0.14 [-0.76, 0.48]
Zehsaz 2016	-0.2	2.52982213	40	0.1	4.15812458	19	-0.09 [-0.64, 0.45]
**1.1.2 Lay vs Standard**							
Jelalian 2015	-0.43	4.03523234	26	-1.28	3.22863748	23	0.23 [-0.34, 0.79]
Kokkvoll 2014	-0.31	0.63592452	36	-0.15	0.50467812	38	-0.28 [-0.73, 0.18]
Looney 2014	-0.08	0.30789609	8	-0.07	0.60893349	7	-0.02 [-1.03, 0.99]
Racil_a 2016	0.4	3.14321414	25	0.3	2.8562029	23	0.03 [-0.53, 0.60]
Shaibi 2006	0.1	5.85576639	16	-0.5	5.07444578	16	0.11 [-0.59, 0.80]
Vos 2011	-0.537	5.13335368	34	-1.148	7.1695731	36	0.10 [-0.37, 0.57]
Wengle_a 2011	0.1	5.85576639	16	-0.5	5.07444578	16	0.11 [-0.59, 0.80]
Wesnigk 2016	0.3	1.07703296	33	1	1.14891253	29	-0.62 [-1.13, -0.11]
**1.1.3 Professional vs Lay**							
Dias 2018	0.03	1.41	95	-0.27	1.2	94	0.23 [-0.06, 0.51]
Hystad 2013	-0.18	0.55434646	36	-0.17	0.45398238	44	-0.02 [-0.46, 0.42]
Jelalian 2010	-1.31	3.26605266	56	-1.5	3.66589689	62	0.05 [-0.31, 0.42]
Johnston 2007	-0.16	1.05	46	0.64	0.9	25	-0.79 [-1.30, -0.29]

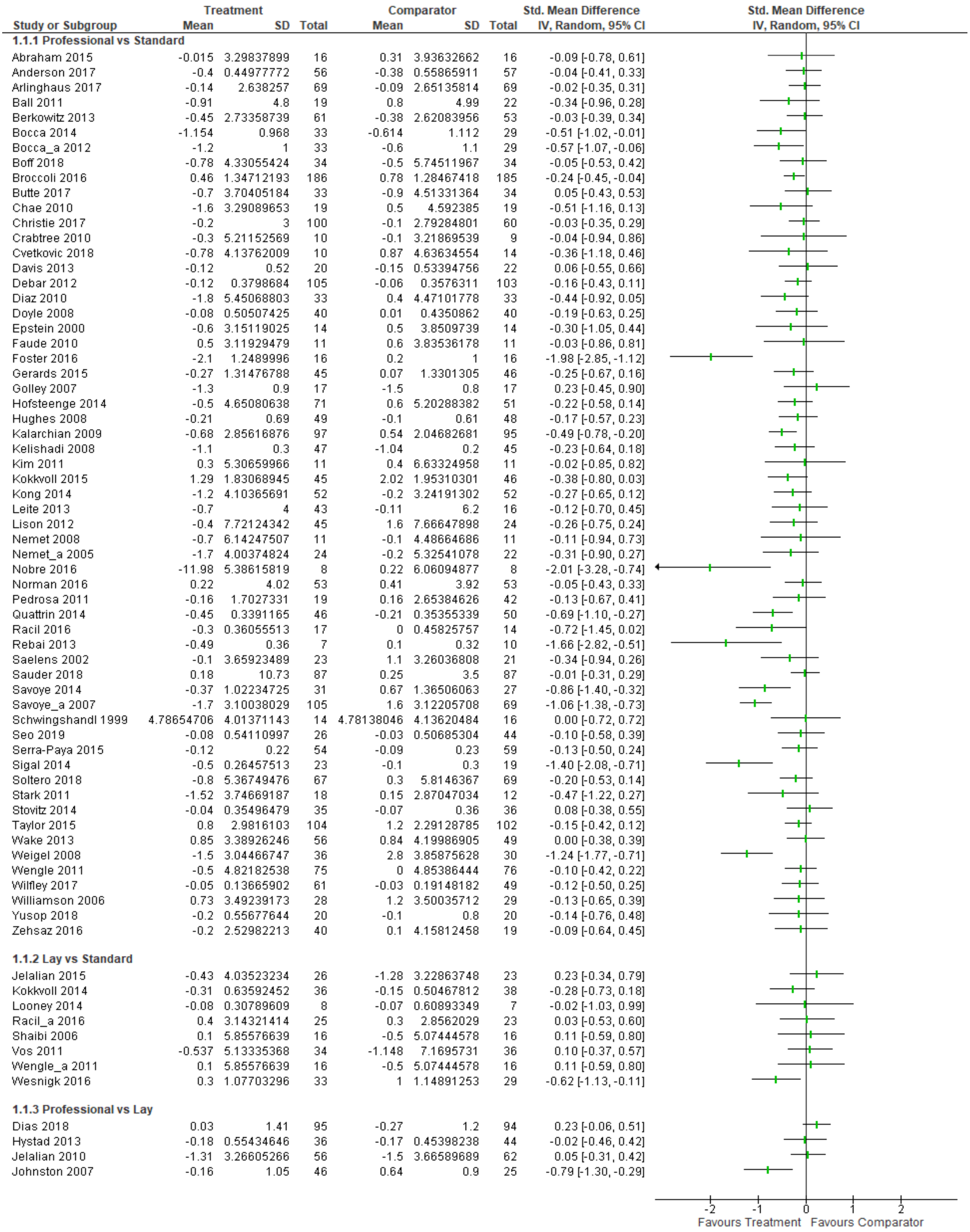

© 2020 McGavock J et al. *JAMA Network Open.*

eFigure 3: Bayesian model inference data for weight change immediately following weight loss intervention

1a. History, Brooks-Gelman-Rubin, Auto correlation and density plots for two primary effect estimates **(Weight)**

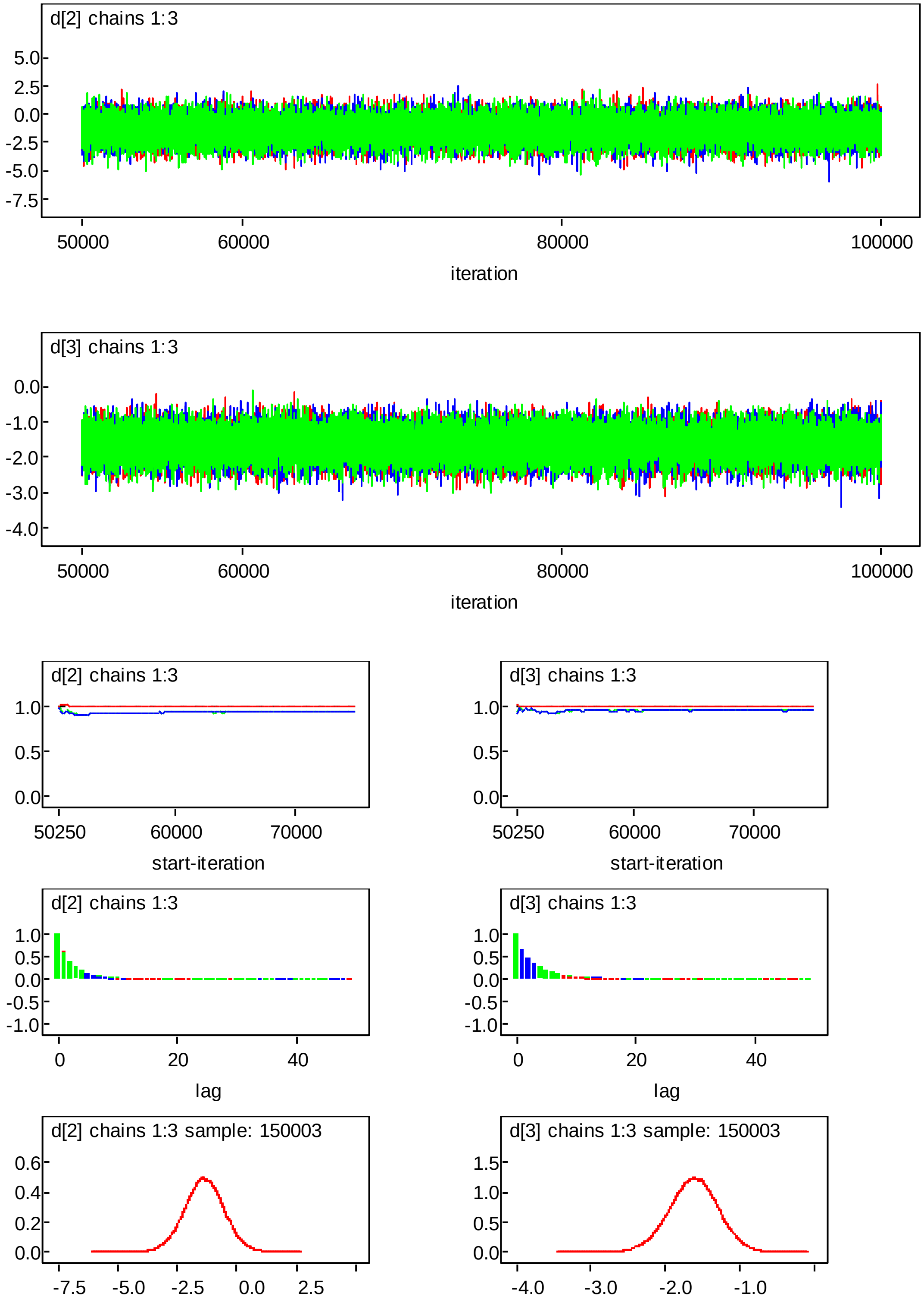

© 2020 McGavock J et al. *JAMA Network Open.*

<u>(BMI)</u>

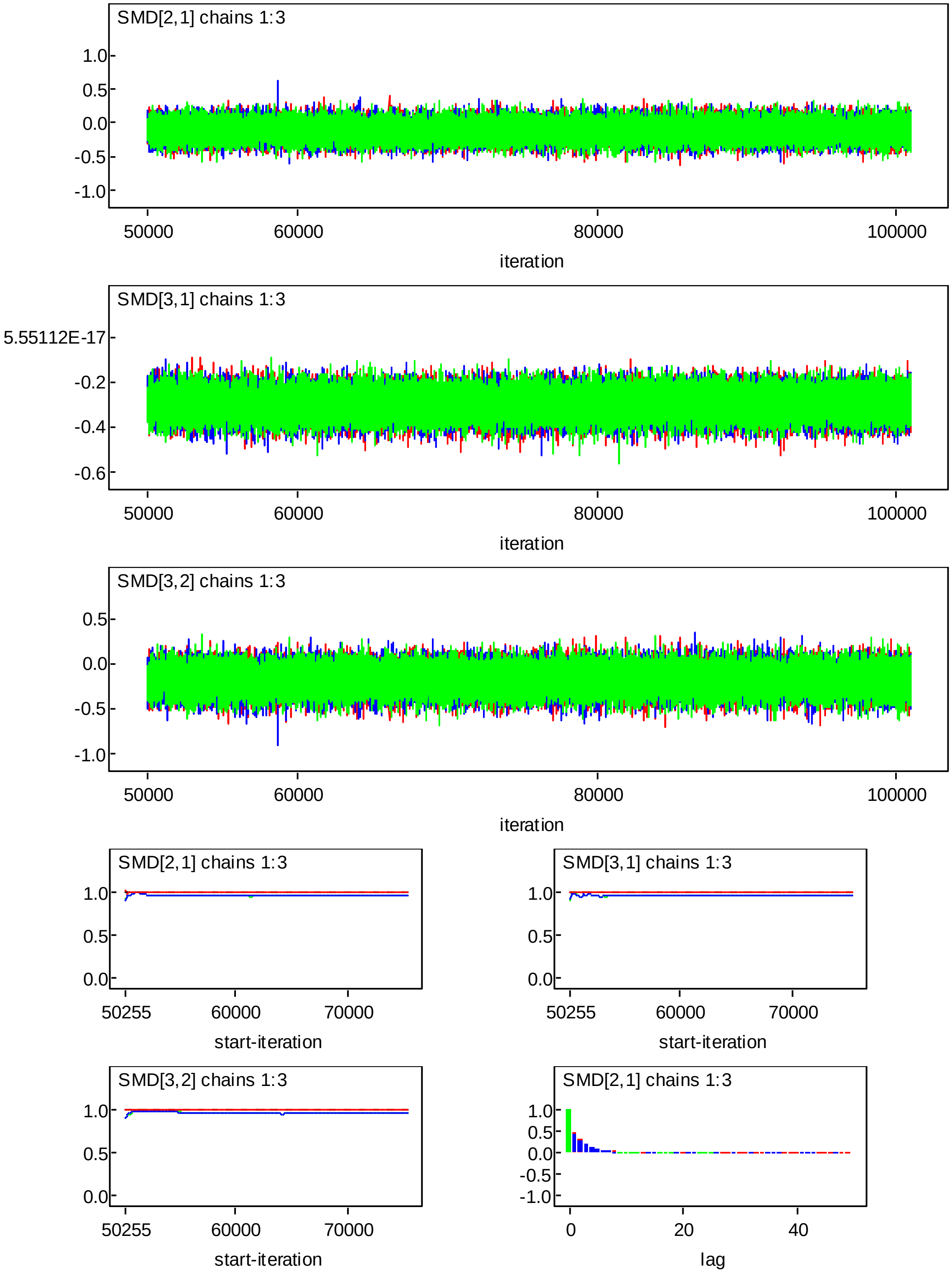

© 2020 McGavock J et al. *JAMA Network Open.*

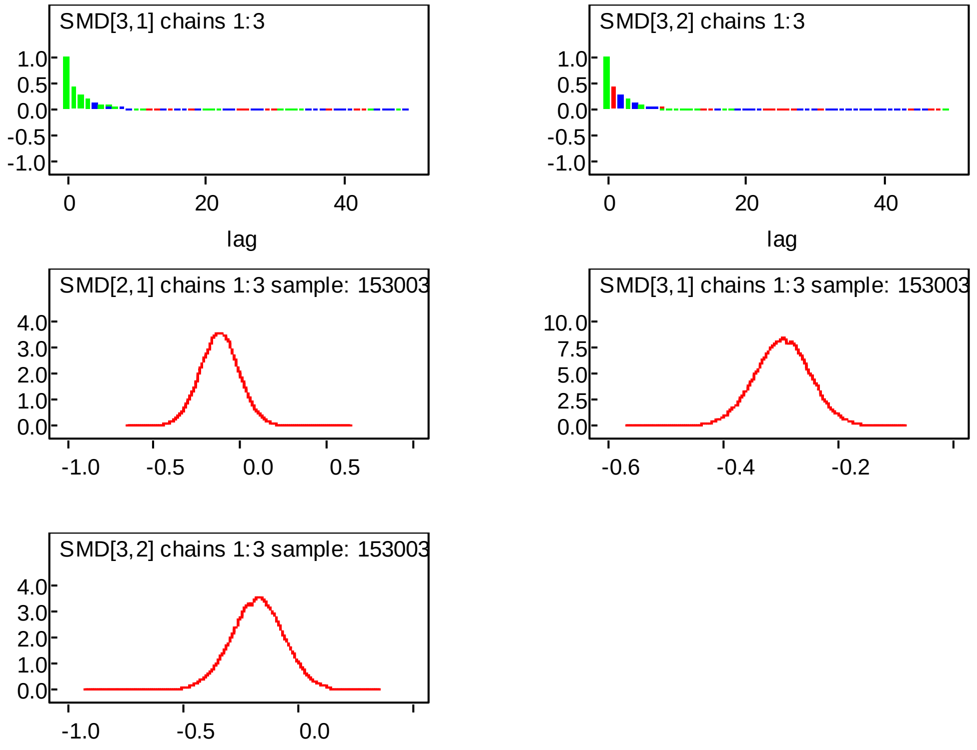

2b. History, Brooks-Gelman-Rubin, Auto correlation and density plots for between study variances for two primary effect estimates

(Weight)

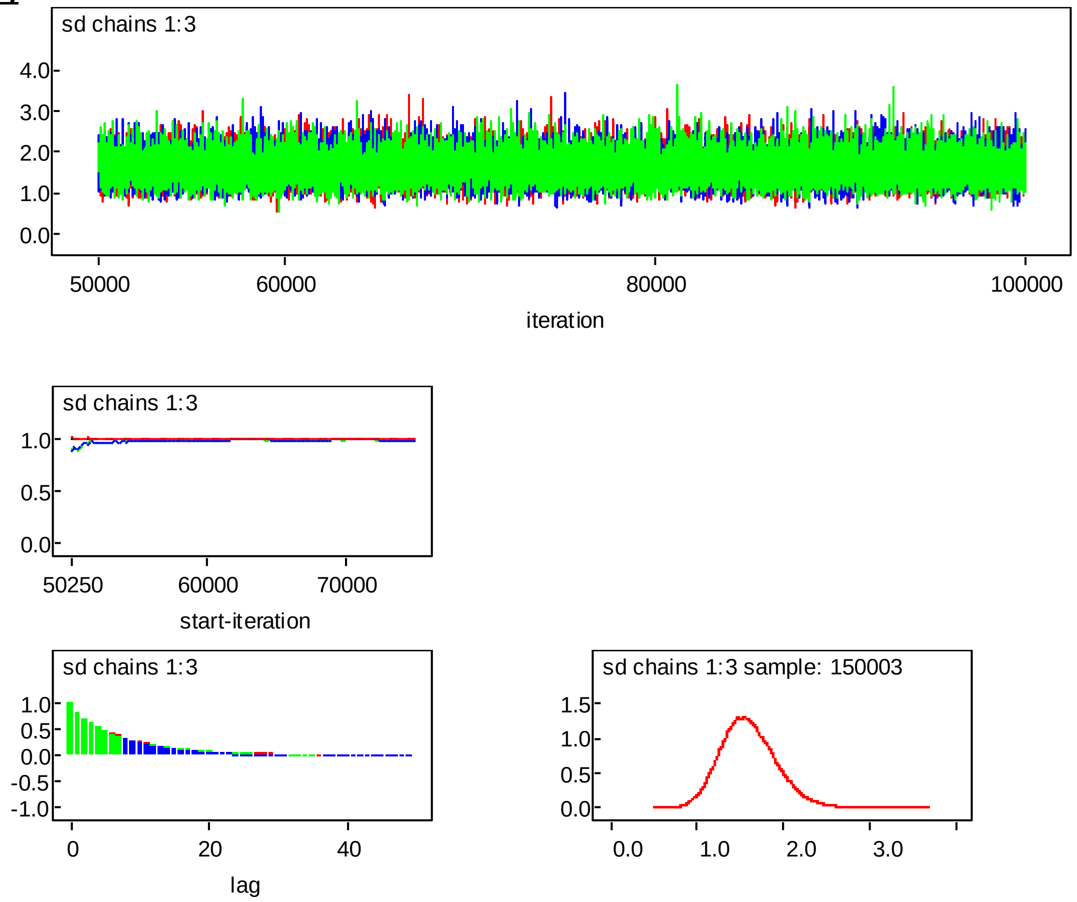

© 2020 McGavock J et al. *JAMA Network Open.*

(BMI)

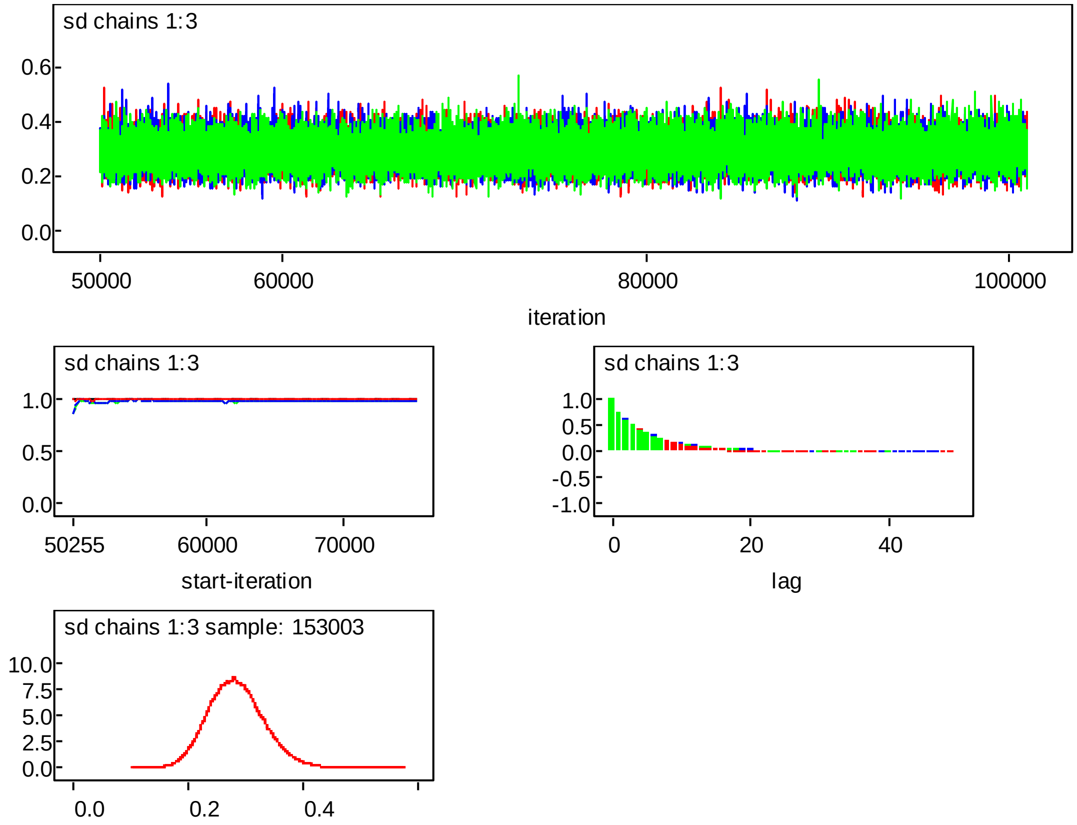

© 2020 McGavock J et al. *JAMA Network Open.*

2c. Residual Deviance Plot:

(Weight)

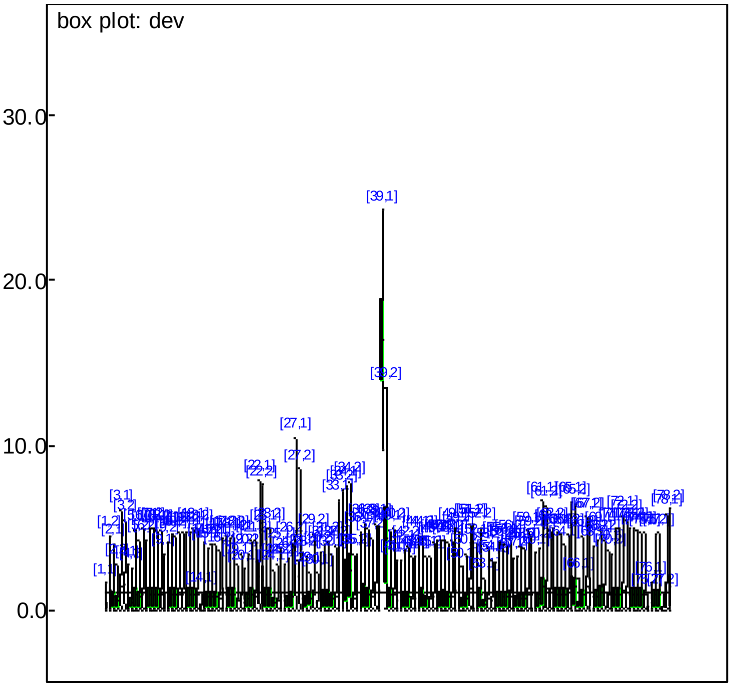

(BMI)

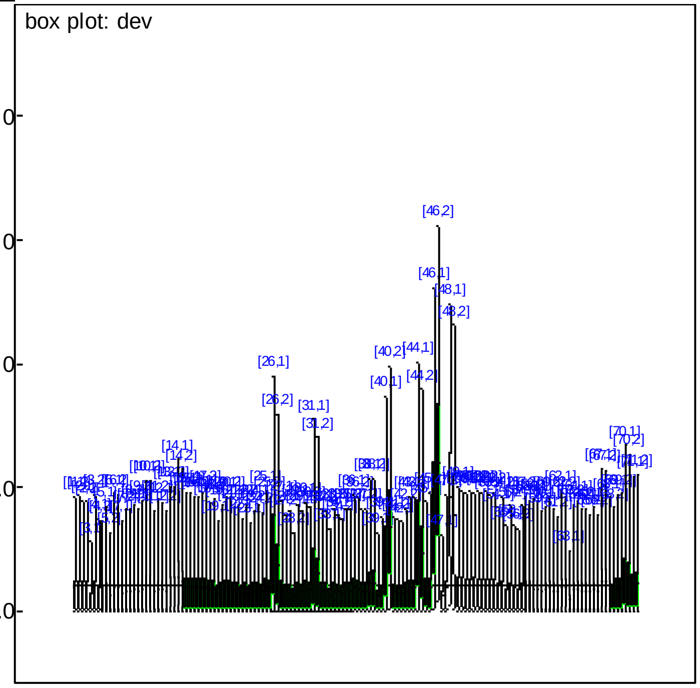

© 2020 McGavock J et al. *JAMA Network Open.*

eFigure 4. Treatment rankings for intervention types for immediate and long-term weight change

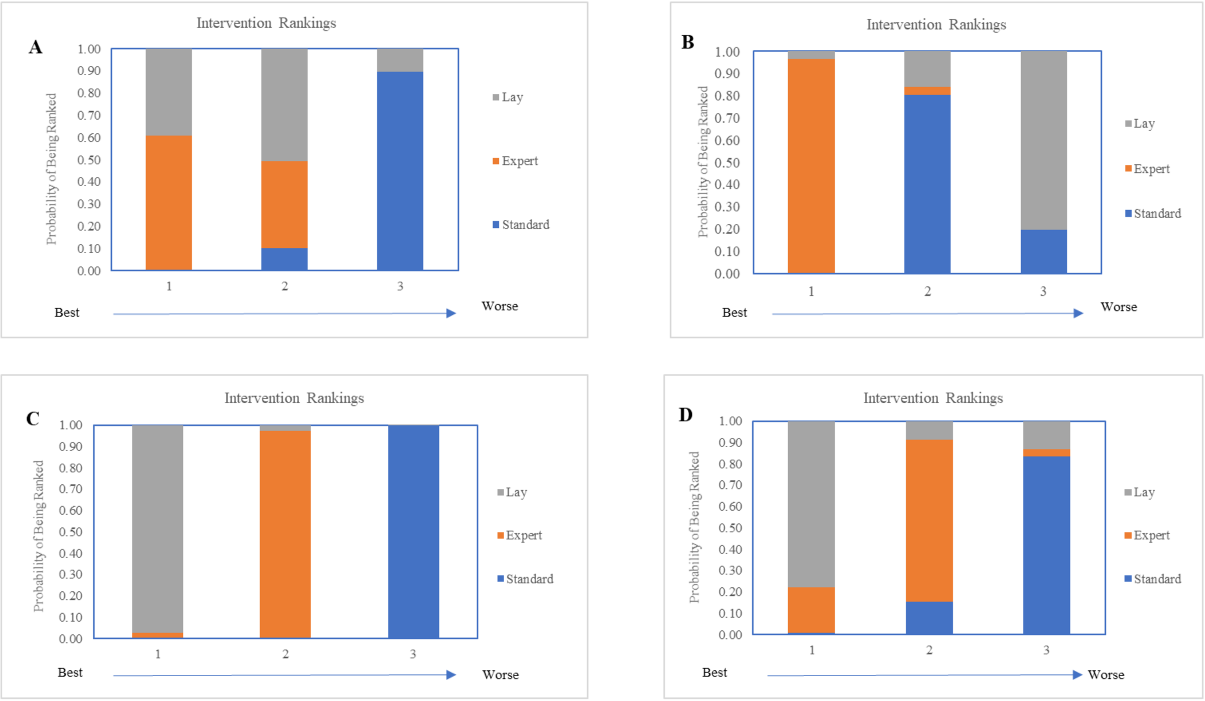

Weight (kg) **BMI (kg/m²)**

SUCRA-based treatment rankings for immediate post intervention (panels A and B) and long-term (panels C and D) change in weight (panels A-C) and change in BMI (panels B-D) obtained from RCTs. Data represent the probability of being ranked as the best (1), next best (2) and least effective (3) intervention. Colours represent the three different intervention types tested in the network.

© 2020 McGavock J et al. *JAMA Network Open.*

eTable 1. Search strategy for Medline.

Database: Ovid MEDLINE from January 1, 1996 to May 20, 2016

1 exp Overweight/ (532946)
2 overweight*.tw,kw. (117790)
3 exp Obesity/ (529073)
4 (obese or obesity).tw,kw. (528645)
5 ((elevat* or excess* or extreme* or heavy or high* or over) adj3 (weight or bodyweight)).tw,kw. (139633)
6 ((elevat* or excess* or extreme* or heavy or high* or over) adj3 (BMI or BMIs or "BMI-Z" or body mass index*)).tw,kw. (51623)
7 or/1-6 (840783)
8 exp Overweight/dh, pc, th (34682)
9 exp Obesity/dh, pc, th (33574)
10 ((prevent* or control* or reduc*) adj3 (obese or obesity or overweight or (weight adj1 gain*) or (bodyweight adj1 gain*))).tw,kw. (54180)
11 Weight Loss/ (124444)
12 ((control* or decreas* or lose or losing or loss or losses or lost or lower* or manag* or modulat* or reduc* or regulat*) adj3 (bodyweight or weight)).tw,kw. (320387)
13 ((control* or decreas* or lower* or manag* or modulat* or reduc* or regulat*) adj3 (BMI or BMIs or "BMI-Z" or body mass index*)).tw,kw. (39583)
14 Caloric Restriction/ (18193)
15 Diet, Carbohydrate-Restricted/ (3007)
16 ((low* or restrict*) adj1 (calori* or calory or carbohydrate* or carb or carbs) adj2 (diet* or eat* or food*)).tw,kw. (10486)
17 Diet, Reducing/ (17797)
18 ((control* or decreas* or lessen* or limit* or lower* or manag* or modulat* or reduc* or regulat* or restrain* or restrict*) adj3 (calori* or calory or eating or food consumption* or food intake* or food crav* or hunger or overeating or over-eating)).tw,kw. (68077)
19 exp Diet Therapy/ (328046)
20 ((diet or nutrition*) adj3 therap*).tw,kw. (20108)
21 Health Education/ (140751)
22 ((health* or nutrition*) adj3 educat*).tw,kw. (117192)
23 exp Health Promotion/ (139808)
24 ((health* or nutrition*) adj3 promot*).tw,kw. (97769)
25 Bright Bodies.tw,kw. (30)
26 Healthy Buddies.tw,kw. (7)
27 (healthy adj2 (diet* or eating or food? or grocer* or lifestyle? or life style? or weight or bodyweight)).tw,kw. (40686)
28 (mindful* adj2 (diet* or eating or food? or grocer* or lifestyle? or life style?)).tw,kw. (190)
29 ((chang* or interven* or modif*) adj3 (lifestyle? or life style?)).tw,kw. (47771)
30 ((chang* or interven* or modif*) adj3 diet*).tw,kw. (68321)
31 exp Exercise Therapy/ (93291)
32 exp Exercise/ (391901)
33 exercis*.tw,kw. (533977)
34 (physical* adj1 activ*).tw,kw. (181043)
35 or/8-34 (1937351)
36 7 and 35 (301731)
37 exp Adult/ not (exp Adult/ and exp Child/) (10923164)

© 2020 McGavock J et al. *JAMA Network Open.*

38 exp Adult/ not (exp Adult/ and Adolescent/) (10059534)
39 exp Adult/ not (exp Adult/ and exp Infant/) (11685961)
40 or/37-39 (11882011)
41 36 not 40 (186476)
42 (controlled clinical trial or randomized controlled trial).pt. (502668)
43 clinical trials as topic.sh. (176725)
44 (randomi#ed or randomly or RCT$1 or placebo*).tw. (1704343)
45 ((singl* or doubl* or trebl* or tripl*) adj (mask* or blind* or dumm*)).tw. (331603)
46 trial.ti. (358168)
47 or/42-46 (2138291)
48 41 and 47 (17231)
49 exp Animals/ not (exp Animals/ and Humans/) (13922259)
50 48 not 49 (10658)
51 (comment or editorial or interview or news or newspaper article).pt. (1656740)
52 (letter not (letter and randomized controlled trial)).pt. (1852289)
53 50 not (51 or 52) (10545)
54 limit 53 to yr="1996-current" (9836)
55 54 use ppez (5566)
56 exp obesity/ (529073)
57 (obese or obesity).tw,kw. (528645)
58 overweight*.tw,kw. (117790)
59 ((elevat* or excess* or extreme* or heavy or high* or over) adj3 (weight or bodyweight)).tw,kw. (139633)
60 ((elevat* or excess* or extreme* or heavy or high* or over) adj3 (BMI or BMIs or "BMI-Z" or body mass index*)).tw,kw. (51623)
61 or/56-60 (839576)
62 exp obesity/dm, pc, th [Disease Management, Prevention, Therapy] (30937)
63 weight reduction/ (152409)
64 ((prevent* or control* or reduc*) adj3 (obese or obesity or overweight or (weight adj1 gain*) or (bodyweight adj1 gain*))).tw,kw. (54180)
65 ((control* or decreas* or lose or losing or loss or losses or lost or lower* or manag* or modulat* or reduc* or regulat*) adj3 (bodyweight or weight)).tw,kw. (320387)
66 ((control* or decreas* or lower* or manag* or modulat* or reduc* or regulat*) adj3 (BMI or BMIs or "BMI-Z" or body mass index*)).tw,kw. (39583)
67 caloric restriction/ (18193)
68 low carbohydrate diet/ (3007)
69 low calory diet/ (8007)
70 ((low* or restrict*) adj1 (calori* or calory or carbohydrate* or carb or carbs) adj2 (diet* or eat* or food*)).tw,kw. (10486)
71 diet therapy/ (58062)
72 ((diet or nutrition*) adj3 therap*).tw,kw. (20108)
73 health education/ (140751)
74 nutrition education/ (3080)
75 ((health* or nutrition*) adj3 educat*).tw,kw. (117192)
76 health promotion/ (137964)
77 ((health* or nutrition*) adj3 promot*).tw,kw. (97769)
78 Bright Bodies.tw,kw. (30)
79 Healthy Buddies.tw,kw. (7)

© 2020 McGavock J et al. *JAMA Network Open.*

80 (healthy adj2 (diet* or eating or food? or grocer* or lifestyle? or life style? or weight or bodyweight)).tw,kw. (40686)
81 (mindful* adj2 (diet* or eating or food? or grocer* or lifestyle? or life style?)).tw,kw. (190)
82 ((chang* or interven* or modif*) adj3 (lifestyle? or life style?)).tw,kw. (47771)
83 ((chang* or interven* or modif*) adj3 diet*).tw,kw. (68321)
84 exp kinesiotherapy/ (57235)
85 exp exercise/ (391901)
86 exercis*.tw,kw. (533977)
87 (physical* adj1 activ*).tw,kw. (181043)
88 or/62-87 (1706927)
89 61 and 88 (271574)
90 exp Adult/ not (exp Adult/ and exp Juvenile/) (5030313)
91 exp Adult/ not (exp Adult/ and exp Child/) (10923164)
92 exp Adult/ not (exp Adult/ and Adolescent/) (10059534)
93 exp Adult/ not (exp Adult/ and exp Infant/) (11685961)
94 or/90-93 (11882011)
95 89 not 94 (165725)
96 randomized controlled trial/ or controlled clinical trial/ (1046936)
97 exp "clinical trial (topic)"/ (192452)
98 (randomi#ed or randomly or RCT$1 or placebo*).tw. (1704343)
99 ((singl* or doubl* or trebl* or tripl*) adj (mask* or blind* or dumm*)).tw. (331603)
100 trial.ti. (358168)
101 or/96-100 (2337898)
102 95 and 101 (18036)
103 exp animal experimentation/ or exp models animal/ or exp animal experiment/ or nonhuman/ or exp vertebrate/ (42278889)
104 exp human/ or exp human experimentation/ or exp human experiment/ (33113683)
105 103 not 104 (9166834)
106 102 not 105 (16433)
107 editorial.pt. (912204)
108 letter.pt. not (letter.pt. and randomized controlled trial/) (1847785)
109 106 not (107 or 108) (16222)
110 limit 109 to yr="1996-current" (15550)
111 110 use oemezd (10518)
112 55 or 111 (16084)
113 limit 112 to yr="2014-current" (5273)
114 remove duplicates from 113 (4470)
115 limit 112 to yr="2011-2013" (5402)
116 remove duplicates from 115 (4524)
117 limit 112 to yr="2006-2010" (3558)
118 remove duplicates from 117 (2814)
119 112 not (113 or 115 or 117) (1851)
120 remove duplicates from 119 (1331)
121 114 or 116 or 118 or 120 (13139) [TOTAL UNIQUE RECORDS]
122 121 use ppez (5432) [MEDLINE UNIQUE RECORDS]
123 121 use oemezd (7707) [EMBASE UNIQUE RECORDS]

© 2020 McGavock J et al. *JAMA Network Open.*

eTable 2. Additional Statistical Methods

Note: Vague priors for treatment effect (d) ~ Normal(0, 10^5) for all model, vague priors for all trial baselines (μ) ~ Normal(0,.0001)

Outcome	Deviance Information Criteria (DIC)	Total Residual Deviance	Bayesian probability of Inconsistency between direct and indirect effect	Vague priors for between trial standard deviation (SD)	Between study heterogeneity (SD) and its credible interval.
**A-Immediate post-intervention weight loss**<br>**Weight**					
Random effect model (used)	725.5	162.7 (with 78*2=156 data points)	0.14	Uniform(0, 5)	1.6 (1.03-2.3)
Fixed effect model	774.5				
**BMI**					
Random effect model (used)	208.2	152.3 (with 71*2=142 data points)	0.96	Uniform(0, 5)	0.28 (0.19 to 0.38)
Fixed effect model	261.8				
**B-Long term weight loss**<br>**Weight**					
Random effect model (Used)	220.9	54.4 (with 26*2=52 data points)	0.14	Uniform(0, 5)	2.0 (1.08 to 3.4)
Fixed effect model	245.8				
**BMI**					
Random effect model (Used)	30.3	50.5 (with 25*2=50 data points)	0.96	Uniform(0, 2)	0.72 (0.52 to 1.03)
Fixed effect model	223.9				

© 2020 McGavock J et al. *JAMA Network Open.*

eTable 3: Baseline characteristics of included randomized controlled trials.

Study ID	N	Gender (% male)			Mean age; Years (SD)			Intervention			Duration of intervention (weeks)	Duration of intervention + follow-up (weeks)	Funding
		Standard	Expert	Lay	Standard	Expert	Lay	Standard	Expert	Lay			
Norman et al. 2015	52	100.0	100.0		11.7 (0.9)	12.0 (0.8)		Enhanced usual care	Physician+nurse+health counselor		52	NA	NR
Norman et al. 2015	54	0.0	0.0		11.8 (1.0)	12.0 (0.9)		Enhanced usual care	Physician+nurse+health counselor		52	NA	NR
Serra-Paya et al. 2015	113	55.9	50.0		9.7 (2.0)	10.1 (2.0)		Counselling	Nereu program (family theoretical counseling+behaviour strategies)		32	NA	Yes
Leite et al. 2013	88	0.0	0.0		12.4 (1.5)	13.1 (1.9)		Nutritional program	Physiologists, nutritionists, physical educators, physicians, and nurses		12	NA	NR
Kulik et al. 2015	41	0.0		0.0	15.1 (1.5)		15.3 (1.5)	Standard care		Group leader, peer	16	NA	Yes
Vos et al. 2011	81	49.0		45.0	13.1 (1.9)		13.3 (2.0)	Physical activity and diet		Dietitian, child physiotherapist, child psychologist, and social worker	12	52	Yes
Wengle et al. 2011	38	31.0		50.0	14.5 (1.4)		14.4 (1.5)	Dietary+behavioural+ physical activity		University student mentor	24	NA	Yes
Ball et al. 2011	31	50.0	41.0		14.8 (1.2)	14.6 (1.3)		Wait list control	Dietician, nurse		16 to 20	NA	Yes
Ball et al. 2011*	15		27.0			16.2 (1.3)		Wait list control	Dietician, nurse		16 to 20	NA	Yes
Crabtree et al. 2010	19	33.0	50.0		11.2 (1.0)	10.9 (1.1)		Usual care	Case manager		12	NA	Yes
Broccoli et al. 2016	372	68.0	75.0		6.5 (1.2)	6.7 (1.0)		Usual care	Psychologists		52	104	NR

© 2020 McGavock J et al. *JAMA Network Open.*

Study ID	N	Gender (% male)			Mean age; Years (SD)			Intervention			Duration of intervention (weeks)	Duration of intervention + follow-up (weeks)	Funding
		Standard	Expert	Lay	Standard	Expert	Lay	Standard	Expert	Lay			
Jelalian et al. 2015	49	35.0		13.0	15.0 (1.3)		15.2 (1.4)	Behavioral treatment		Parents	16	NA	Yes
Abraham et al. 2015	32	62.0	56.0					Usual care+internet-based curriculum	Physician		24	NA	Yes
Abraham et al. 2015*	16		62.0					Usual care+internet-based curriculum	Dietician/nutritionist		24	NA	Yes
Stark et al. 2014	28	33.0	36.0		4.8 (0.7)	4.2 (1.1)		Dietary/physical activity instructions	Clinical psychologist, psychology postdoctoral fellow		26	52	Yes
Stark et al. 2014*	14		20.0			4.7 (1.3)		Dietary/physical activity instructions	Clinical psychologist, psychology postdoctoral fellow		26	52	Yes
Kokkvoll et al. 2015	97	52.0	40.0		10.5 (1.7)	10.1 (1.7)		Single-family intervention	Paediatric nurse, paediatric consultant, and nutritionist		104	NA	Yes
Bocca et al. 2014	75	25.7	30.0		4.7 (0.8)	4.6 (0.8)		Usual care	Dietician, physiotherapist, pediatrician		16	156	Yes
Looney et al. 2014	22	62.5	14.3	14.3	7.3 (1.8)	8.6 (1.8)	8.2 (1.8)	Usual care	Trained interventionist		26	NA	Yes
Looney et al. 2014*								Usual care	Pediatrician		26	NA	Yes
Love-Osborne et al. 2014	165	54.0	42.0		16.0 (1.5)	15.7 (1.5)		Motivational interviewing	Health educator		24 to 32	NA	Yes
Quattrin et al. 2014	105	34.0	32.6		4.4 (1.5)	4.6 (1.4)		Diet and activity education	Practice enhancement assistants		52	104	Yes
Hamilton-Shield et al. 2014	61	40.0	50.0		9.6 (1.9)	9.1 (1.6)		Standard care	Nurse		NR	NA	Yes
Hofsteenge et al. 2014	122	41.2	46.5		14.4 (1.8)	14.5 (1.7)		Standard care	Dietician, paediatrician/endocrinologist, and psychologist		24	76	Yes

© 2020 McGavock J et al. *JAMA Network Open.*

Study ID	N	Gender (% male)			Mean age; Years (SD)			Intervention			Duration of intervention (weeks)	Duration of intervention + follow-up (weeks)	Funding
		Standard	Expert	Lay	Standard	Expert	Lay	Standard	Expert	Lay			
Kong et al. 2014	104	46.2	40.4		16.7 (1.0)	16.8 (1.0)		Usual diet	Dietitian		52	78	Yes
Bocca et al. 2014a	75	25.7	30.0		4.7 (0.8)	4.6 (0.8)		Usual care	Dietitian, physiotherapist, and psychologist		16	NA	Yes
Stovitz et al. 2014	72	53.0	46.0		5.7 (1.5)	6.2 (1.6)		Usual care	Trained research assistant		12	NA	Yes
Savoye et al. 2014	75	37.8	31.6		13.2 (1.8)	12.7 (1.9)		Standard care	Dietitian, psychologist, exercise physiologist/physical therapist		24	NA	Yes
Berkowitz et al. 2013	92	19.1	24.4		14.3 (1.5)	14.5 (1.5)		Lifestyle modification programs	Nurses, nurse practitioners, dietitians, counselors, psychologists		52	NA	Yes
Berkowitz et al. 2013*	77	26.8	22.2		14.9 (1.4)	14.7 (1.3)		Lifestyle modification programs	Nurses, nurse practitioners, dietitians, counselors, psychologists		52	NA	Yes
Davis et al. 2013	58	70.4	71.0		8.7 (1.8)	8.5 (1.7)		Physician visit	Trained PHD-level psychologists or trained graduate students/postdoctoral fellows		32	NA	Yes
Hystad et al. 2013	99		48.9	51.9		9.9 (1.5)	10.5 (1.9)		Psychologists, paediatricians, clinical dietitians and physiotherapists	Parents	104	NA	Yes
Wake et al. 2013	118	59.0	50.0		7.4 (2.2)	7.2 (2.3)		Usual care	Paediatrician, dietitian		52	NA	Yes
Bocca et al. 2012	75	25.7	30.0		4.7 (0.8)	4.6 (0.8)		Standard behavioral treatment	Dietician, physiotherapist, psychologist		16	52	Yes

© 2020 McGavock J et al. *JAMA Network Open.*

Study ID	N	Gender (% male)			Mean age; Years (SD)			Intervention			Duration of intervention (weeks)	Duration of intervention + follow-up (weeks)	Funding
		Standard	Expert	Lay	Standard	Expert	Lay	Standard	Expert	Lay			
Lison et al. 2012	110	54.0	51.0	49.0	11.2 (10.3)	12.3 (13.6)	11.9 (14.1)	Diet and lifestyle changes	Pediatricians, nutritionist, physical education instructor	Parents	26	NA	Yes
Debar et al. 2012	208	0.0	0.0		14.0 (1.5)	14.1 (1.5)		Usual care	Master's level nutritionists, health educators, and doctoral level clinical psychologists		20	52	Yes
Kalavainen et al. 2012	70							Usual care	Dietitian		26	NA	Yes
Pedrosa et al. 2011	83							Conventional treatment	Nutritionist		52	NA	Yes
Chae et al. 2010	38	52.6	57.9		10.6 (16.6)	10.4 (13.5)		Lifestyle modification counselling	Educated instructors		12	NA	NR
Jelalian et al. 2010	118		31.0	34.0		14.2 (0.9)	14.5 (1.1)		Dietician, master- and doctoral-level psychologists	Peer-enhanced adventure therapy	16	52	Yes
Ellis et al. 2010	49							Multisystemic therapy (intensive, family-centered, community-based treatment)	Dietician, therapists		26	NA	Yes
Faude et al. 2010	39	72.7	54.5					Standard exercise program	Experienced students of physical education		26	NA	Yes
Berntsen et al. 2010	60							Dietary/physical activity advice	Experienced instructors		20	NA	Yes
Diaz et al. 2010	76	50.0	48.0		11.7 (2.2)	11.6 (2.1)		Primary care physicians consultation	Dietitian, physician		52	NA	Yes
Kalarchian et al. 2009	192	42.1	44.3		10.3 (1.2)	10.1 (1.2)		Usual care	Lifestyle coach		26	78	Yes
Weigel et al. 2008	73	50.0	40.5		11.6 (2.0)	10.9 (1.4)		Therapeutic advice from physician	Sports coaches, dietitians, and psychologists		52	NA	Yes

© 2020 McGavock J et al. *JAMA Network Open.*

Study ID	N	Gender (% male)			Mean age; Years (SD)			Intervention			Duration of intervention (weeks)	Duration of intervention + follow-up (weeks)	Funding
		Standard	Expert	Lay	Standard	Expert	Lay	Standard	Expert	Lay			
Kelishadi et al. 2008	100							Exercise	Paediatrician, nutritionist, and dietician		26	52	Yes
Nemet et al. 2008	22	36.0	36.0		10.2 (1.7)	9.9 (1.0)		Usual care	Dietitian, professional youth trainer		12	NA	Yes
Doyle et al. 2008	83	40.0	35.0		14.1 (1.6)	14.9 (1.7)		Usual care	Pediatricians and nurses		16	32	Yes
Hughes et al. 2008	134	44.6	43.4		8.5 (1.9)	9.1 (1.7)		Diet	Pediatric dietitians		26	52	Yes
Johnston et al. 2007	71		47.8	40.0		12.2 (0.8)	12.2 (0.7)		Instructor/trainer-led intervention	Parents	26	104	Yes
Savoye et al. 2007	174	31.8	43.8		12.4 (2.3)	11.9 (2.5)		Diet	Exercise physiologists		52	NA	Yes
Williamson et al. 2006	57							Nutrition education	Counselor		104	NA	Yes
Jelalian et al. 2006	89					14.7 (1.0)	14.3 (0.8)	Standard Care	Nutritionist, doctoral level psychologists, exercise physiologist or physical therapist	Peer-enhanced adventure therapy	16	40	NR
Epstein et al. 2005	41	47.4	40.9		10.1 (1.3)	10.2 (1.1)		Standard behavioral treatment	Not clearly reported		104	NA	Yes
Balagopal et al. 2005	15	57.0	50.0		15.9 (0.5)	15.6 (0.3)		usual care + advice on physical activity and diet	Pediatrician and/or nurse practitioner		12	NA	Yes
Nemet et al. 2005	46	55.0	58.0		11.3 (2.8)	10.9 (1.9)		Nutritional consultation	Professional youth coaches, dietitian		12	52	Yes
Saelens et al. 2002	44							Usual care	Pediatrician, telephone counselors		16	28	Yes
Schwingshandl et al. 1999	30	44.0	43.0		12.2 (0.7)	11 (2.5)		Usual care	Dietitian		12	NA	NR
Taylor et al. 2015	206	45.0	44.0		6.4 (1.4)	6.5 (1.4)		Usual care	Dietitian, exercise specialist, and clinical psychologist		104	NA	Yes

© 2020 McGavock J et al. *JAMA Network Open.*

Study ID	N	Gender (% male)			Mean age; Years (SD)			Intervention			Duration of intervention (weeks)	Duration of intervention + follow-up (weeks)	Funding
		Standard	Expert	Lay	Standard	Expert	Lay	Standard	Expert	Lay			
Gerards et al. 2015	86	45.2	43.2		7.3 (1.3)	7.14 (1.6)		Brochure and knowledge quiz via email	Health professionals		16	52	Yes
Kokkvoll et al. 2014	97	52.2	40.0		10.5 (1.7)	10.1 (1.7)		Usual care	Paediatric and psychiatric nurse, paediatric consultant, nutritionist, physiotherapist, coach, and clinical educationalist		12	52	Yes
Gesell et al. 2010	159	64.0	46.0		9.7 (1.1)	9.6 (1.1)		Usual care+counselling	Program manager		26	NA	Yes
Golley et al. 2007	74	36.0		37.0				Healthy-lifestyle pamphlet		Parents	26	52	NR
Golley et al. 2007*	37		35.0					Healthy-lifestyle pamphlet	Physical activity experts		26	52	NR
Epstein et al. 2000	34	47.1	47.1		10.0 (1.2)	10.7 (0.9)		Behavioral weight-control program	Therapist		26	130	Yes
Epstein et al. 2000*	18		50.0			10.3 (1.2)		Behavioral weight-control program	Therapist		26	130	Yes
Hofsteenge et al. 2013	95	36.8	49.1		14.5 (1.7)	14.6 (1.6)		Usual care	Dietician, psychologist, and paediatric-endocrinologist		26	78	Yes
Regaieg et al. 2013	28	72.7	72.7		10.6 (0.7)	10.9 (0.6)		Exercise	Cardiologist		16	NA	NR
Stark et al. 2011	18	60.0	75.0		3.9 (1.1)	4.4 (0.9)		Counselling	Pediatrician, clinical psychologist, pediatric psychology postdoctoral fellows and a research coordinator		26	52	Yes
Barkin et al. 2011	159							Usual care	Counseling by a physician, trained bilingual study member		26	NA	Yes

© 2020 McGavock J et al. *JAMA Network Open.*

Study ID	N	Gender (% male)			Mean age; Years (SD)			Intervention			Duration of intervention (weeks)	Duration of intervention + follow-up (weeks)	Funding
		Standard	Expert	Lay	Standard	Expert	Lay	Standard	Expert	Lay			
Kim et al. 2011	30	100.0	100.0					Regular physical education	Professional aerobic dancer		12	NA	Yes
Wesnigk et al. 2016	16				14.9 (3.6)	15.0 (2.5)		Waitlist control, usual care and counselling	Qualified physiotherapist		43.4	NA	Yes
Anderson et al. 2017	203	42.0	51.0		10.3 (3.2)	10.7 (3.0)		Usual care	Physical activity coordinator, dietician, psychologist		52	NA	Yes
Yusop et al. 2018	50	50.0	55.0		9.8 (1.2)	9.8 (1.2)		Nutritional counselling	Researcher, dietician, professional exercise instructor		26	NA	Yes
Shaibi et al. 2006	28				15.6 (0.5)	15.1 (0.5)		Usual care	Exercise specialist		16	NA	Yes
Sigal et al. 2014	151	31.6	29.3		15.6 (1.3)	15.5 (1.4)		Waitlist control	Exercise specialist		22	NA	Yes
Sigal et al. 2014*	154	31.6	29.5		15.6 (1.3)	15.9 (1.5)		Waitlist control	Exercise specialist		22	NA	Yes
Sigal et al. 2014*	151	31.6	29.3		15.6 (1.3)	15.5 (1.3)		Waitlist control	Exercise specialist		22	NA	Yes
Cvetkovic et al. 2018	28				Range 11-13	Range 11-13		Waitlist control	Research staff		12	NA	NR
Cvetkovic et al. 2018*	28	NR	NR		Range 11-13	Range 11-13		Waitlist control	Research staff		12	NA	NR
Seo et al. 2019	103	61.0	63.0		12.4 (2.1)	12.9 (1.7)		Usual care	Dietician, physician		16	NA	Yes
Racil et al. 2016	42	0.0	0.0		16.9 (1.0)	16.6 (0.9)		Usual care	Experienced physical education teacher		12	NA	Yes
Racil et al. 2016*	45	0.0	0.0		16.9 (1.0)	16.5 (1.2)		Usual care	Experienced physical education teacher		12	NA	Yes
Wilfley et al. 2017	116	42.0	37.0		9.5 (1.3)	9.5 (1.3)		Weight management education	Dietician, research staff		17.4	34.8	Yes
Wilfley et al. 2017*	113	42.0	36.0		9.5 (1.3)	9.4 (1.2)		Weight management education	Dietician, research staff		17.4	34.8	Yes

© 2020 McGavock J et al. *JAMA Network Open.*

Study ID	N	Gender (% male)			Mean age; Years (SD)			Intervention			Duration of intervention (weeks)	Duration of intervention + follow-up (weeks)	Funding
		Standard	Expert	Lay	Standard	Expert	Lay	Standard	Expert	Lay			
Soltero et al. 2018	136	51.0	40.2		15.3 (0.9)	15.4 (1.0)		General information	Fitness instructors		13.0	52	Yes
Racil et al. 2016	31	0.0	0.0		14.2 (1.2)	14.2 (1.2)		NR	NR		12	NA	Yes
Racil et al. 2016*	30	0.0	0.0		14.2 (1.2)	14.2 (1.2)		NR	NR		12	NA	Yes
Christie et al. 2017	174	37.0	38.0		15.0	15.0		Enhanced standard care by nurse	Psychology graduates		26	52	Yes
Yackobovitch-Gavan et al. 2018	180	34.0	35.0		8.1 (1.3)	8.7 (1.5)		Usual care	Psychology, dietician		13	104	Yes
Yackobovitch-Gavan et al. 2018*	180	34.0	24.0		8.1 (1.3)	8.5 (1.7)		Usual care	Psychology, dietician		13	104	Yes
Butte et al. 2017	160	49.5	49.5		Range 2-5	Range 2-5		Usual care (Next Steps)	Dieticians, health educators		13	52	Yes
Butte et al. 2017*	181	49.5	49.5		Range 6-8	Range 6-8		Usual care (Next Steps)	Dieticians, health educators		13	52	Yes
Butte et al. 2017*	208	49.5	49.5		Range 9-12	Range 9-12		Usual care (Next Steps)	Dieticians, health educators		13	52	Yes
Dias et al. 2018	67	46.1	48.5		11.8 (2.4)	12.4 (1.9)		Dietician advice	Research staff		12	NA	Yes
Dias et al. 2018*	66	46.1	46.9		11.8 (2.4)	11.9 (2.4)		Dietician advice	Research staff		12	NA	Yes
Boff et al. 2018	135	41.7	58.3		16.4 (1.2)	16.5 (1.0)		Traditional health education, and multidisciplinary team (psychology, physiotherapy, nursing)	Interdisciplinary team (psychology, physiotherapy, nursing)		12	NA	Yes
Zehsaz et al. 2016	32	100.0	100.0		10.3 (0.9)	10.8 (0.9)		Usual care	Exercise specialists		16	NA	No
Nobre et al. 2017	59	NR	NR		9.9 (1.1)	9.8 (0.9)		NR	Research staff		12	NA	Yes
Hay et al. 2016	74	36.0	13.0		15.2 (1.7)	15.3 (1.7)		Waitlist control	Research staff, kinesiologist		24	NA	Yes

© 2020 McGavock J et al. *JAMA Network Open.*

Study ID	N	Gender (% male)			Mean age; Years (SD)			Intervention			Duration of intervention (weeks)	Duration of intervention + follow-up (weeks)	Funding
		**Standard**	**Expert**	**Lay**	**Standard**	**Expert**	**Lay**	**Standard**	**Expert**	**Lay**			
Hay et al. 2016*	68	36.0	22.0		15.2 (1.7)	15.1 (1.8)		Waitlist control	Research staff, kinesiologist		24	NA	Yes
Sauder et al. 2018	62	58.0		45.0	9.1 (1.1)		9.3 (1.0)	Educational control	Lay health coaches		34.8	NA	Yes
Wengle et al. 2011	38	31.2		50.0	14.4 (1.5)		14.4 (1.4)	NR	University student mentor		26	NA	Yes
Foster et al. 2016	60	50.0		63.0	4.3 (0.6)		4.5 (0.7)	Community meetings	Parent mentors		26	52	Yes
Arlinghaus et al. 2017	189		48.0	49.0		12.9 (0.6)	12.9 (0.5)	Physical education teacher	Physical education teacher and high school mentors		26	52	Yes

Data are presented as mean (SD). * indicates RCT compared more than two groups. BMI= Body mass index.

© 2020 McGavock J et al. *JAMA Network Open.*

eTable 4. Cochrane risk of bias assessment for eligible RCTs.

Study	Random sequence generation	Allocation concealment	Blinding of participants and personnel	Incomplete outcome data	Selective reporting	Other bias	Overall Judgement
Norman et al. 2015	Low	Unclear	High	Low	Unclear	Low	High
Serra-Paya et al. 2015	Low	Unclear	Low	Low	Unclear	Low	Low
Leite et al. 2013	Unclear	Unclear	High	High	Low	High	High
Kulik et al. 2015	Low	Low	Unclear	Low	Unclear	Low	Low
Vos et al. 2011	Unclear	Unclear	High	Low	Low	Low	High
Wengle et al. 2011	Low	Unclear	High	Low	Low	Low	High
Ball et al. 2011	Unclear	Low	Low	Low	Low	Low	Low
Crabtree et al. 2010	Unclear	Unclear	High	Unclear	Unclear	Unclear	High
Broccoli et al. 2016	Low	Unclear	High	Low	Low	Low	High
Jelalian et al. 2015	Low	Unclear	High	Low	Low	Low	High
Abraham et al. 2015	Low	Low	High	Unclear	Low	Low	High
Stark et al. 2014	Low	Unclear	High	Low	Low	Low	High
Kokkvoll et al. 2015	Unclear	Unclear	Low	Low	Low	Low	Low
Bocca et al. 2014	Unclear	Unclear	High	Low	Low	Low	High
Looney et al. 2014	Unclear	Low	High	Low	Low	Low	High
Love-Osborne et al. 2014	Unclear	Unclear	High	Low	Unclear	Low	High
Quattrin et al. 2014	Low	Unclear	High	Low	Low	Low	High
Hamilton-Shield et al. 2014	Low	Low	High	Low	Unclear	Low	High
Hofsteenge et al. 2014	Low	Unclear	High	Low	Low	Low	High
Kong et al. 2014	Low	Low	High	Low	Low	Low	High
Bocca et al. 2014a	Unclear	Unclear	High	Low	Unclear	Low	High
Stovitz et al. 2014	Unclear	Unclear	Low	Low	Unclear	Low	Low
Savoye et al. 2014	Low	Unclear	Low	Low	Low	Low	Low
Berkowitz et al. 2013	Low	Unclear	High	Low	Low	Low	High

© 2020 McGavock J et al. *JAMA Network Open.*

Davis et al. 2013	Low	Unclear	High	High	Low	Low	High
Hystad et al. 2013	Low	Unclear	High	Low	Low	Low	High
Wake et al. 2013	Low	Unclear	Low	Low	Low	Low	Low
Bocca et al. 2012	Low	Unclear	High	Low	Low	Low	High
Lison et al. 2012	High	Unclear	Low	Low	Unclear	Low	High
Debar et al. 2012	Low	Unclear	Low	Low	Low	Low	Low
Kalavainen et al. 2012	Unclear	Unclear	High	Unclear	Unclear	Low	High
Pedrosa et al. 2011	Unclear	Unclear	High	Low	Low	Low	High
Chae et al. 2010	Unclear	Unclear	High	Unclear	Unclear	Low	High
Jelalian et al. 2010	Low	Unclear	High	Low	Low	Low	High
Ellis et al. 2010	Unclear	Unclear	High	Low	Unclear	Low	High
Faude et al. 2010	Unclear	Unclear	Low	High	Unclear	Low	High
Berntsen et al. 2010	Low	Unclear	High	Unclear	Unclear	Low	High
Diaz et al. 2010	Low	Low	Low	Low	Low	Low	Low
Kalarchian et al. 2009	Low	Unclear	High	Low	Low	Low	High
Weigel et al. 2008	Unclear	Unclear	High	Low	Low	Low	High
Kelishadi et al. 2008	Low	Unclear	High	Low	Unclear	Low	High
Nemet et al. 2008	Low	Unclear	High	Low	Unclear	Low	High
Doyle et al. 2008	Unclear	Low	Low	Low	Low	Low	Low
Hughes et al. 2008	Low	Low	Low	Low	Low	Low	Low
Johnston et al. 2007	Unclear	Unclear	High	Low	Low	Low	High
Savoye et al. 2007	Unclear	Unclear	High	Low	Low	Low	High
Williamson et al. 2006	Low	Unclear	High	Low	Low	Low	High
Jelalian et al. 2006	Low	Unclear	High	Low	Low	Low	High
Epstein et al. 2005	Unclear	Unclear	High	Low	Unclear	Low	High
Balagopal et al. 2005	Unclear	Unclear	High	Unclear	Unclear	Low	High
Nemet et al. 2005	Low	Unclear	High	Low	Unclear	Low	High
Saelens et al. 2002	Unclear	Low	High	Low	Low	Low	High
Schwingshandl et al. 1999	Unclear	Unclear	High	Unclear	Unclear	Low	High
Taylor et al. 2015	Low	Low	Low	Low	Low	Low	Low

© 2020 McGavock J et al. *JAMA Network Open.*

Gerards et al. 2015	Low	Low	Low	Low	Low	Low	Low
Kokkvoll et al. 2014	Low	Low	Low	Low	Low	Low	Low
Gesell et al. 2010	Unclear	Unclear	High	High	Low	Low	High
Golley et al. 2007	Low	Low	Low	Low	Low	Low	Low
Epstein et al. 2000	Unclear	Unclear	High	Unclear	Unclear	Low	High
Hofsteenge et al. 2013	Low	Unclear	High	Low	Low	Low	High
Regaieg et al. 2013	Unclear	Unclear	High	Unclear	Unclear	Low	High
Stark et al. 2011	Low	Unclear	High	Low	Low	Low	High
Barkin et al. 2011	Unclear	Unclear	High	Low	Low	Low	High
Kim et al. 2011	Unclear	Unclear	High	Unclear	Unclear	Low	High

Low= High methodological quality; High= Low methodological quality; Unclear= Inadequate information was reported to judge.

© 2020 McGavock J et al. *JAMA Network Open.*

eTable 5. Mean and median probabilities of treatment ranks

Outcome	Professional-led	Layperson-led	Standard
Immediate Follow-up			
**Weight (kg)**			
Mean Rank (±SD)	1.38 (±0.48)	1.67 (±0.56)	2.95 (±0.21)
Median Rank (95% CrI)	1 (1, 2)	2 (1, 3)	3 (2, 3)
**BMI (kg/m^2)**			
Mean Rank (±SD)	1.06 (±0.24)	2.08 (±0.45)	2.86 (±0.03)
Median Rank (95% CrI)	1 (1, 2)	2 (1, 3)	3 (2, 3)
Long-term follow-up			
**Weight (kg)**			
Mean Rank (±SD)	1.55 (±0.56)	1.74 (±0.79)	2.70 (±0.53)
Median Rank (95% CrI)	2 (1, 2)	2 (1, 3)	3 (1, 3)
**BMI (kg/m^2)**			
Mean Rank (±SD)	1.67 (±0.07)	2 (±0.90)	2.33 (±0.07)
Median Rank (95% CrI)	2 (1, 3)	2 (1, 3)	2 (1, 3)

© 2020 McGavock J et al. *JAMA Network Open.*
